# CK2β-regulated signaling controls B cell differentiation and function

**DOI:** 10.3389/fimmu.2022.959138

**Published:** 2023-01-11

**Authors:** Laura Quotti Tubi, Elisa Mandato, Sara Canovas Nunes, Arash Arjomand, Fortunato Zaffino, Sabrina Manni, Alessandro Casellato, Paolo Macaccaro, Nicola Vitulo, Sara Zumerle, Odile Filhol, Brigitte Boldyreff, Christian W. Siebel, Antonella Viola, Giorgio Valle, Federica Mainoldi, Stefano Casola, Valeria Cancila, Alessandro Gulino, Claudio Tripodo, Marco Pizzi, Angelo Paolo Dei Tos, Livio Trentin, Gianpietro Semenzato, Francesco Piazza

**Affiliations:** ^1^ Department of Medicine, Division of Hematology, University of Padova, Padova, Italy; ^2^ Unit of Normal and Malignant Hematopoiesis, Laboratory of Myeloma and Lymphoma Pathobiology, Veneto of Molecular Medicine (VIMM), Padova, Italy; ^3^ Department of Medical Oncology, Dana-Farber Cancer Institute, Boston, MA, United States; ^4^ Division of Hematology/Oncology, Boston Children's Hospital, Harvard Medical School, Boston, MA, United States; ^5^ Department of Biology, Interdepartmental Research Center for Biotechnologies (CRIBI) Biotechnology Center, University of Padova, Padova, Italy; ^6^ Department of Biomedical Sciences, University of Padova, Padova, Italy; ^7^ Institut National de la Santé Et de la Recherche Médicale (INSERM) U1036, Institute de Recherches en Technologies et Sciences pour le Vivant/Biologie du Cancer et de l’Infection, Grenoble, France; ^8^ Kinase Detect ApS, Krusaa, Denmark; ^9^ Department of Discovery Oncology, Genentech, Inc., South San Francisco, CA, United States; ^10^ IFOM-ETS-The AIRC Institute of Molecular Oncology, Milan, Italy; ^11^ Tumor Immunology Unit, University of Palermo, Palermo, Italy; ^12^ Department of Medicine, Cytopathology and Surgical Pathology Unit, University of Padova, Padova, Italy

**Keywords:** B lymphocyte, B cell development, protein kinase CK2, marginal zone, germinal center, Diffuse large B cell lymphoma, B cell receptor signaling

## Abstract

Serine-Threonine kinase CK2 supports malignant B-lymphocyte growth but its role in B-cell development and activation is largely unknown. Here, we describe the first B-cell specific knockout (KO) mouse model of the β regulatory subunit of CK2. CK2β^KO^ mice present an increase in marginal zone (MZ) and a reduction in follicular B cells, suggesting a role for CK2 in the regulation of the B cell receptor (BCR) and NOTCH2 signaling pathways. Biochemical analyses demonstrate an increased activation of the NOTCH2 pathway in CK2β^KO^ animals, which sustains MZ B-cell development. Transcriptomic analyses indicate alterations in biological processes involved in immune response and B-cell activation. Upon sheep red blood cells (SRBC) immunization CK2β^KO^ mice exhibit enlarged germinal centers (GCs) but display a limited capacity to generate class-switched GC B cells and immunoglobulins. *In vitro* assays highlight that B cells lacking CK2β have an impaired signaling downstream of BCR, Toll-like receptor, CD40, and IL-4R all crucial for B-cell activation and antigen presenting efficiency. Somatic hypermutations analysis upon 4-Hydroxy-3-nitrophenylacetyl hapten conjugated to Chicken Gamma Globulin (NP-CGG) evidences a reduced NP-specific W33L mutation frequency in CK2β^KO^ mice suggesting the importance of the β subunit in sustaining antibody affinity maturation. Lastly, since diffuse large B cell lymphoma (DLBCL) cells derive from GC or post-GC B cells and rely on CK2 for their survival, we sought to investigate the consequences of CK2 inhibition on B cell signaling in DLBCL cells. In line with the observations in our murine model, CK2 inactivation leads to signaling defects in pathways that are essential for malignant B-lymphocyte activation.

## 1 Introduction

In B lymphocytes intra- and intercellular signaling pathways are instrumental for cell fate commitment and acquisition of effector functions (1). Studies in the mouse have shown that two mature B cell subsets form the splenic white pulp, follicular (Fo) B cells that home in the follicles but can recirculate through the bone marrow (BM) upon activation and marginal zone (MZ) B cells that reside around the follicles at the edge between white and red pulp (2). The commitment of transitional type 2 (T2) B cells towards a Fo or a MZ B cell fate depends on the strength of the B cell receptor (BCR) signaling and on the activation of the NOTCH2 pathway (3). The “signal strength” model supports the concept that a strong BCR signal, which blocks the activation of NOTCH2, promotes Fo B cell development. After antigen (Ag) encounter, Fo B cells give rise to germinal centers (GCs), eventually generating long-lived memory B cells or plasma cells (PCs) homing to the BM (4). During the process of malignant B lymphocyte transformation, several genetic and epigenetic alterations can occur affecting B cell signaling (5). Components of the BCR cascade like CD79B and CARD11 or of the Toll-like Receptor (TLR) signaling, like MYD88, are mutated at high frequency in B-cell lymphomas (5–8). Besides genetic alterations, lymphoma B-cell growth is controlled by constitutively active protein kinases as demonstrated by the successful therapeutic strategy targeting Bruton’s Tyrosine Kinase (BTK) or Phosphoinositide 3 kinase (PI3K) in non-Hodgkin lymphomas (9). The Ser/Thr kinase CK2 is a tetramer composed of two catalytic α and two regulatory β subunits. These latter are required for substrate recruitment and kinase activity modulation (10). CK2 has been found overexpressed in a wide array of solid and hematologic tumors (11–15) and shown to sustain survival and proliferation of malignant B cells in chronic lymphocytic leukemia, multiple myeloma, mantle cell lymphoma, B-acute lymphoblastic leukemia and diffuse large B cell lymphoma (DLBCL) (13, 16–22). Multiple proteins taking part in hemo-lymphopoiesis are CK2 substrates, however, the physiological role of this kinase in B cell biology has been poorly explored. A recent work has shown that conditional knockout (KO) of the catalytic CK2α subunit in B cells causes a perturbed peripheral B cell differentiation characterized by an accumulation of MZ B cells driven by NOTCH2 (23).

To investigate the function of the regulatory β subunit of CK2 in B lymphocytes, we generated CK2β KO mice in CD19-expressing cells. Our data have highlighted increased NOTCH2 activation and impaired BCR signaling in CK2β-deficient cells, which skew splenic B cells towards a MZ rather than a Fo commitment. Intriguingly, CK2β-deficient B cells form larger germinal centers (GCs) and are defective in generating class-switched GC B cells. We show that CK2β exerts a major function in BCR, TLR, IL-4R and CD40 signaling pathways, impinging on multiple cascades, as its loss leads to a significantly reduced phosphorylation of BTK, NF-κB, AKT, and ERK1/2 upon receptors engagement. Of note, AKT-dependent FOXO1 phosphorylation as well as NF-κB and ERK1/2-mediated upregulation of AID, IRF4 and BCL6 are impaired. Consequently, CK2β-deficient B cells are unable to acquire full competency to generate an effective GC reaction and undergo class-switch recombination (CSR) and affinity maturation.

Our findings establish CK2β as a pivotal controller of peripheral B cell commitment and function with implications in B cell physiology and pathobiology.

## 2 Materials and methods

### 2.1 Mouse strains

Mice of the C57BL/6 and C57BL/6 CD19-Cre strains were purchased from Jackson Laboratories, USA. Mice carrying a loxP-flanked *Csnk2b* allele (*Csnk2b^fl^*) were provided by B. Boldyreff. To obtain *Csnk2b^fl/fl^*; CD19-Cre (CK2β^κO^) mice, *Csnk2b^wt/fl^* (24) and CD19-Cre mice were intercrossed. *Csnk2b^+/+^*; CD19-Cre mice were used as controls (CK2β^CTRL^). Mice were bred and housed in a pathogen–free colony at the animal facility of the Venetian Institute of Molecular Medicine (VIMM), Padova. The University of Padova Organism in charge for animal wellness (OPBA) approved the experimentation and declared that it fulfilled the National and European rules for animal studies. All the experimental procedures were authorized by the Italian Ministry of Health (Prot. number 126/2015 and Prot. 205/2022). Mice were sacrificed at 8-12 weeks to perform experiments.

### 2.2 Cell purification

Spleen and lymph nodes (LN) were disrupted and filtered from cell debris through a 70 μm cell strainer to prepare cell suspensions. BM cells were isolated from hind limbs. Bones were flushed with PBS + 2% FCS and filtered through a 70 μm cell strainer. Peripheral blood (PB) was obtained from the retro orbital venous plexus. Erythrocytes were removed using a lysis buffer (BD). B cells were purified by negative selection using EasySep™ Mouse B Cell Isolation Kit (Stemcell). Fo and MZ B cell subpopulations were purified with FACSAria (BD) using CD23-PE (B3B4), CD21-APC (7G6), B220/CD45R-APC-Cy7 (RA3-6B2), CD19-PerCP-Cy5.5 (1D3), all BD. After immunization with NP-CGG, GC B cells (B220^high^, IgD^-^, CD38^low/-^, PNA^+^, CD95^+^) were sorted by FACSAria (BD) using B220/CD45R-APC-Cy7 (RA3-6B2), IgD-V450, CD38-PE, peanut agglutinin (PNA-FITC) and CD95-PE-Cy7 (Jo2).

### 2.3 Flow cytometry

The following Abs were used: CD19-FITC (1D3), CD5-FITC (53-7.3), IgD-FITC (11-26c.2a), B220/CD45R-FITC (RA3-SB2), CD19-PE (1D3), IgM-PE (R6-60.2), CD184(CXCR4)-PE (2B11), CD138-PE (281-2), CD23-PE (B3B4), CD95(Fas)-PE-Cy7 (Jo2), CD19-PerCP-Cy5.5 (1D3), Streptavidin-PerCP-Cy5.5, CD25-APC (PC61), B220/CD45R-APC (RA3-6B2), CD21-APC (7G6), IgG1-APC (A85-1), CD86-APC (GL1), IgM-PE-Cy7 (R6-60.2), c-Kit(CD117)-PE-Cy7 (2B8), B220/CD45R-APC-Cy7 (RA3-6B2), IgD-V450 (11-26c.2a), biotin-IgG3 (R40-82) (all BD), CD27-AF647 (LG3A10) (BioLegend), *Lectin from Arachis hypogaea*-FITC (Sigma). Data were acquired on a FACSCanto or FACSAria cytometer and analyzed using FACSDiva 6.0 (BD) software. Cells were stained in PBS for 10 min at room temperature (RT) with Abs to the appropriate markers, preceded by the blockade of Fc receptors with CD16/CD32 Fc block (2.4G2) (BD) for 5 min at 4°C. Staining for BrdU was done as follows: after surface staining, cells were washed, resuspended in Cytofix/Cytoperm buffer (BD), incubated on ice for 20 min, washed with Perm/Wash buffer (BD) and incubated in Cytoperm permeabilization Buffer (BD) for 10 min on ice. After that, cells were washed with Perm/Wash buffer and incubated with DNase I (Sigma, 300μg/ml) in PBS plus Ca^++^ and Mg^++^ for 1 hour at 37°C. After washing, cells were incubated with FITC-conjugated anti-BrdU (BD) at RT for 30 min.

### 2.4 Absolute cell counts by flow cytometry

To determine the absolute number of B cells in peripheral blood, spleen and bone marrow we used the volumetric method in flow cytometry (25). In detail, 100µl of whole blood (with EDTA) was stained with anti-B220 antibody and then diluted in 2ml of lysis buffer; 200µl of sample were transferred into 96 well U bottom plate and read immediately in duplicate by FacsCanto using the same settings of flow rate (1µl/s) and sample volume (150µl) for all the samples analyzed. The absolute count of B220^+^ cells/µl was obtained using the formula: events recorded/150µl * dilution factor.

A similar approach and reading parameters were used also for spleen and bone marrow: total spleen and a smaller fraction of this organ were subsequently weighted. The smaller piece was smashed in PBS and filtered keeping a fixed resuspension volume of 2ml. For the bone marrow, one femur was processed for each mouse and bone flushing with PBS was followed by bone crushing and filtering keeping a fixed volume of 2ml. 200µl of this suspension were transferred into a 96 well V bottom plate, centrifuged, resuspended in 50µl of supernatant, and stained with anti-B220 antibody without lysing. Wash with 150µl of PBS and resuspended in 200µl final volume. Absolute counts were expressed as events in the whole spleen or femur.

### 2.5 Histology, immunohistochemistry and *in situ* mRNA hybridization

Samples were fixed in formalin and embedded in paraffin; 4 μm-thick sections were stained with H&E. The frequency of GCs was calculated by manually counting the number of GCs on spleen sections and dividing this value by the total spleen section area, in a blinded manner (four mice per genotype). The ratio MZ/lymphoid follicle area was esteemed as previously described (26). Image acquisition was performed using Leica DMD108 Digital Microimaging Device and Software (Leica Microsystems, Germany). Pictures were acquired using 4x, 10x, 20x and 40x objectives at RT. IHC analysis was performed using an automated platform (Bond-maX; Leica, Newcastle Upon Tyne, UK). Tissue sections were treated with the Bond Dewax Solution (Leica) at 72°C. The CPS/EDTA or Heat/EDTA Ag retrieval methods were used, according to the manufacturer’s instructions. Abs: polyclonal BCL6 (Santa Cruz). *AICDA* transcript (ID: 11628) was detected using RNAscope 2·5 HD Detection Reagent-BROWN (Advanced Cell Diagnostic) in accordance with the manufacturer’s protocol. Quantitative analyses of *Aicda* mRNA *in situ* hybridization signals were performed by calculating the average percentage of positive signals in five nonoverlapping fields at high-power magnification (X400) using the Positive Pixel Count v9 ImageScope software, Leica Biosystems.

### 2.6 Immunofluorescence

Spleens were fixed with 4% paraformaldehyde for 3 hours, washed with PBS and left overnight in 20% sucrose to dehydrate. Eight μm thick-sections were blocked with 5% BSA in PBS, stained with surface Abs, washed and blocked with 5% BSA plus 0.3% Triton X-100 in PBS, stained with intracellular Abs in 0.3% Triton X-100 in PBS and mounted with a Fluoromount Aqueous Mounting Medium (Sigma) without DAPI. Abs used: FITC anti-CD169 (MOMA1; AbD Serotec), PE anti-IgM (R6-60.2, BD), V450 anti-IgD (R6-60.2; BD). Images were acquired with Zeiss LSM 700 confocal microscope and analyzed with ZEN software. Pictures were acquired using 10x/0.3 dry and 20x/0.8 dry objectives at RT and merged in three-color images with ImageJ.

### 2.7 mRNA preparation and sequencing

Total RNA samples were processed according to Dynabeads^®^ mRNA DIRECT^™^ Purification Kit (Ambion) to isolate mRNA. The template library was prepared using the Ion Total RNA-Seq Kit v2 (Life Technologies). Quantity and size distribution of the library were analyzed using the Agilent Bioanalyzer 2100 DNA HS chip. Emulsion PCR using 10μl of 100pM library was performed using a OneTouch 2 instrument (Life Technologies) with an Ion PI Template OT2 200 kit following manufacturer’s instructions. The enrichment of template library was achieved using the Ion OneTouch ES enrichment system (Life Technologies). Ion Proton sequencer and IPv2 chip were prepared according to the manufacturer’s recommendations.

### 2.8 Reads mapping and identification of differentially expressed genes

Sequencing adapters were trimmed using cutadapt software (27) and reads shorter than 16bp were removed. Reads were aligned on the reference mouse genome (GTCm38/mm10 version) downloaded from Ensembl (http://www.ensembl.org/) using a two-step procedure. At first, reads were aligned using the STAR program (28). All reads that did not align were realigned with bowtie (29) using a local alignment strategy. Gene expression was quantified using the Ensembl gene annotation, version 75, with htseq-count program (http://www-huber.embl.de/users/anders/HTSeq/doc/count.html). The row count matrix was normalized to consider the GC content biases and the different coverage depth. Normalization was performed using the full quantile normalization implemented EDASeq R package (30); the differentially expression analysis with the EdgeR package (31). Genes with a p value lower or equal 0.05 after false discovery rate correction were considered significantly differentially expressed.

To determine whether an *a priori* defined set of genes shows statistically significant, concordant differences between the two phenotypes, the normalized GSE89082 matrix was used for gene set enrichment analysis (GSEA) by doing 1000 gene set permutations and weighted signal2noise statistical analysis.

### 2.9 qRT-PCR

RNA was extracted with the RNeasy mini kit (Qiagen) and reverse transcribed using the Reverse Transcription System (Promega). qRT-PCR was performed on the ABI PRISM 7000 Sequence Detection System (Applied Biosystems) using the FastStart Universal SYBR GREEN master (ROX) (Roche) or with QuantStudio 5 (Thermofisher) using Luna Universal qPCR Master Mix (New England BiLabs). Data were analyzed with the ABI PRISM 7000 software (Applied Biosystems) and with on-line tools on the Applied Biosystems platform (Thermo Fisher cloud); mRNA levels were normalized on the expression of *Gapdh/Actin*. Results are presented *as ratio* between normalized expression of the gene of interest in the target and in the calibrator samples. The complete list of primers is reported in **Supplemental Table 1**.

### 2.10 Amplification of V_H_1-72 rearrangements from splenic GC B cells

NP-specific V-gene analysis was performed as described in (32). PCR primers used for amplification of the **V_H_** 1-72 rearrangements are reported in **Supplemental Table 2**. Briefly, genomic DNA and RNA were extracted from sorted GC B cells with RNeasy AllPrep MicroKit (QIAGEN) following the manufacturer’s protocol. For **V_H_** 1-72 gene amplification 2 rounds of PCR amplification were performed using 5µl Pfu Buffer (10x), 1µl Pfu polymerase, 1µl dNTPs 10mM, 1ul of each primer (V_H_186.2 Fw and JH2 Rv for the first round; V_H_186.2 inner primer Fw and JH2 Rv for the second round), water and DNA to reach 50µl final volume. After initial denaturation at 95° for 2’30’’, samples were subjected to 20 (the first PCR round) or 30 cycles (the second PCR round) of PCR amplification (30’’ at 95°C, 30 s at 70°C, and 1’30’’ at 72°C) with final elongation for 5’ at 72°C. To inactivate Pfu polymerase, second-round PCR products were incubated for 30’ at -20°C. PCR products were gel-purified and cloned into pGEM-T easy vector (Promega). Plasmids containing inserts were subjected to Sanger sequencing. IgV gene mutational analysis was performed using IgBlast software.

### 2.11 Cell lysis and Western Blot (WB)

Five-7 x 10^6^ cells were prepared by lysis in a buffer made up of 20 mM Tris, 150 mM NaCl, 2 mM EDTA (ethylenediaminetetraacetic acid), 2 mM EGTA (ethylene glycol-tetra-acetic acid), 0.5% v/v Triton X-100 supplemented with protease inhibitor cocktail (Sigma), 1 mM DTT (dithiothreitol; Amersham Biosciences), 1 mM PMSF (phenyl-methyl-sulfonyl fluoride; Sigma), 1 mM okadaic acid (Sigma) and phosphatase inhibitor cocktail (Thermo Scientific). Proteins were subjected to SDS-PAGE, transferred to PVDF membranes and immunoblotted with the following primary Abs: anti-CK2α (provided by Dr. M. Ruzzene, University of Padova, Italy), anti-CK2β, anti-RELA, anti-FOXO1 (Abcam), anti-pRELA S529 (recognizes S527 in mouse), anti-IRF4, anti-BLIMP-1, anti-BCL6 (Santa Cruz), anti-GAPDH (Ambion), anti-pAKT S129 (provided by Dr. M. Ruzzene, University of Padova, Italy), anti-AID (Invitrogen), anti-NOTCH2 (D76A6), anti-pAKT S473, anti-AKT, anti-pERK1/2 T202/Y204, anti-pBTK Y223, anti-ERK1/2, anti-pPTEN S380/T382/T383, PTEN, anti-pFOXO1 S256, anti-pSTAT6 Y705, anti-STAT6 (Cell Signaling). Secondary Abs: anti-rabbit IgG HRP-linked Ab (Cell Signaling), HRP labeled goat anti-mouse IgG (KPL), goat anti-rat IgG HRP-conjugated (Calbiochem), donkey anti-goat IgG HRP-conjugated (Santa Cruz).

### 2.12 B cell culture

Splenic B cells were cultured (10^6^ cells/ml) in RPMI (Euroclone) + 10% FCS (Euroclone) and 2-mercaptoethanol (50 mM; Life Technologies) for 72h with 20 μg/ml LPS (Sigma) ± 10 ng/ml IL-4 (Sigma) or for 48h with 10 μg/ml anti-CD40 (BD Pharmingen) ± 10 ng/ml IL-4 (Sigma). To perform fast stimulation 5x10^6^ B cells were resuspended in 400ul PBS without FCS and treated for 5 and 10 minutes with 10μg/ml anti-CD40 (BD Pharmingen) ± 10 ng/ml IL-4 or for 1, 5, 10 minutes with 10 μg/ml anti-Mouse IgM (Jackson Immuno Research); the incubation was performed in water bath at 37°C followed by the addiction of ice-cold PBS to stop the stimulation; cells were immediately pelleted and lysed. DLBCL cell lines OCI-Ly1, OCI-Ly18 and Pfeiffer were purchased from the Deutsche Sammlung von Mikroorganismen und Zellkulturen (DSMZ, Germany). OCI-Ly1 were maintained in IMDM (Invitrogen) + 20% FBS + 2-Mercaptoethanol (50 mM); OCI-Ly18 in RPMI + 10% FBS + 2-Mercaptoethanol (50 mM); Pfeiffer in RPMI + 10% FBS + Sodium Pyruvate 1 mM. The cells were maintained in an incubator at 37°C in a modified atmosphere with 5% CO2. Testing for Mycoplasma infection was carried out on a monthly basis. All procedures of handling were carried out under a sterile hood.

### 2.13 *In vivo* immunization

Mice were immunized intraperitoneally with 200 μl of SRBC (Microbiol) diluted 1:10 in PBS or with 100 μl of Keyhole Limpet Hemocyanin (NP-CGG) (Biosearch Technologies) previously dissolved in PBS and mixed at 1:1 ratio with Alum (Thermo Scientific). After 14 days, PB was withdrawn to perform ELISA and spleens were removed for immunohistochemical and cytometric analysis.

### 2.14 Continuous Brdu labeling

Mice were given sterile drinking water containing 0.5 mg/ml BrdU (Sigma) plus 1% sucrose for 6 days. BrdU-containing drinking water was changed after 3 days.

### 2.15 *In vivo* inhibition of NOTCH2 activation

Mice were injected intraperitoneally with 15 mg/kg α-NRR2, provided by Dr. C.W. Siebel (Genentech, CA, USA) or IgG (Jackson ImmunoResearch Laboratories, Inc.). After 72 hours from the injection of a single dose of α-NRR2, mice were sacrificed to analyze the spleen.

### 2.16 *In vitro* CK2 kinase activity assay

CK2 activity in whole cell lysates was measured using Casein Kinase 2 Assay Kit (Millipore) with a CK2β-specific substrate peptide, provided by Prof. O. Marin (University of Padova, Italy). Cell lysates (2.5 μg) were incubated with the peptide (200 μM) in ADBI buffer, according to the manufacturer’s instructions. Counts per minute (Cpm) of enzyme samples were normalized to those of control samples containing no peptide. CK2β-specific peptide sequence: MSGDEMIFDPTMSK_8_P(NH_2_)

### 2.17 Enzyme linked immunosorbent assay

For measurements of Ig production after immunization with SRBC or B cell stimulation with LPS ± IL-4, ELISA assays were performed on mouse serum or cell culture supernatant with Mouse Immunoglobulin Isotyping ELISA kit (BD), according to the manufacturers’ instructions. To evaluate the immune response after NP-CGG immunization, a direct ELISA test was performed to measure IgG1 production against NP-23 and NP-4 antigens; detailed information about this procedure can be found in **Supplemental Material**.

### 2.18 Analysis of Ca^2+^ mobilization

Splenic B cells pretreated or not with CX-4945 (Sellekchem) 5μM for 3 hours, were loaded with Fluo4-AM (4μg/ml; Molecular Probes) and FuraRed (16μg/ml; Molecular Probes), sulfinpyrazone (250μM; Sigma) and Pluronic F-127 (0,1% w/v, Life Technologies) in RPMI for 30 min at 37°C in the dark. Cells were washed with RPMI and resuspended in warm PBS at the density of 10^6^ cells/ml. After recording for 30 sec to establish the baseline, cells were stimulated with 20 μg/ml F(ab’)_2_ rabbit anti–mouse IgM (Jackson ImmunoResearch Laboratories, Inc.) for 6.5 min. Then, ionomycin (2 μg/ml) was added to allow the complete emptying of Ca^++^ stores. Measurements were monitored using a FACSCanto cytometer and analyzed using FlowJo software (Tree Star).

### 2.19 IP_3_ uncaging

B cells were seeded on poly-L-lysine (100 μg/ml, Sigma) coated coverslips (10^5^ cells) and loaded with caged-IP_3_ (1 μM; Enzo Life Science), Fluo-4-AM (0,5 μM; Molecular Probes), Pluronic F-127 (0,1% w/v; Life Technologies), sulfinpyrazone (250 μM; Sigma) in RPMI without FCS for 30 min at 37°C. Live calcium imaging was performed in HBSS using a Zeiss LSM700 laser scanning confocal microscope. Time-lapse images were acquired for 1 min (0,6 frame/sec), using a 40x water immersion objective (Zeiss W Plan-Apochromat 40x/1.0 DIC M27). Fluo-4-AM was excited with a 488-nm laser (0,5% power). Regions of interest (ROIs) were drawn on four distinct cells per field; the UV laser (405 nm, 100% power, 10 ms pulse) was used to release active IP_3_ within the ROIs. Images were analyzed using ImageJ software. Fluo-4 traces were generated by averaging pixel signals within the ROIs and normalized on the baseline fluorescence of the first five frames (ΔF/F_0_).

### 2.20 Statistical analysis

Data were evaluated for their statistical significance with the two-tailed Student’s *t* test. Values were considered significant at p value below 0.05. Prism 6 (GraphPad Software) was used for analyses.

### 2.21 Accession number

The complete RNA-seq data are available at the Gene Expression Omnibus (http://www.ncbi.nlm.nih.gov/geo) under accession number GSE89082.

## 3 Results

### 3.1 Loss of CK2β causes a reduction in recirculating B cells and hypogammaglobulinemia

**Figure 1:**
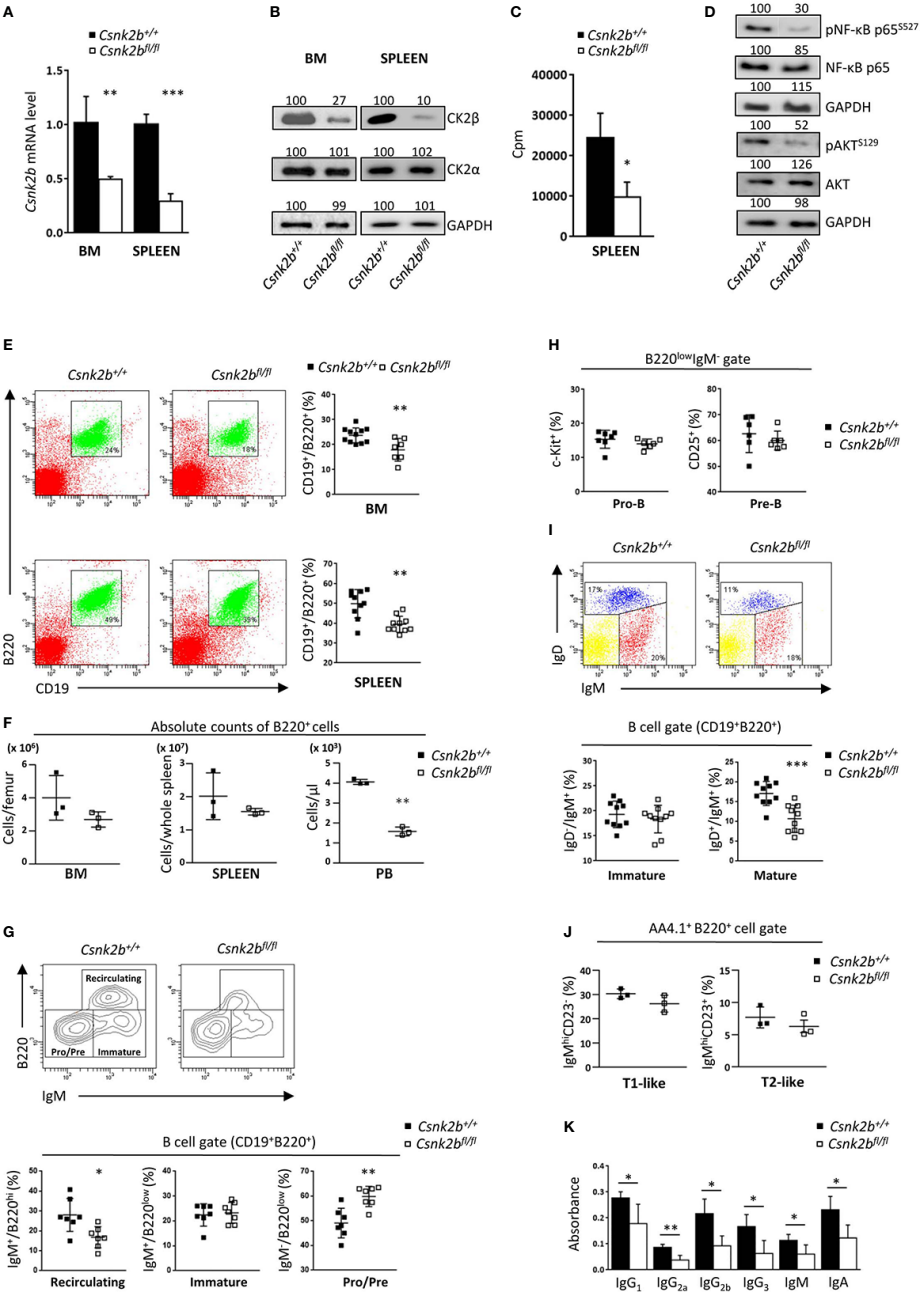
CK2β KO in B cells affects B cell number and Igs production. **(A)** CD19^+^ sorted marrow and splenic B cells analyzed for the expression of *Csnk2b* by qRT-PCR. The expression was corrected for *Actin* levels and normalized to CTRL B cells. Data are shown as mean ± SD (n=4 BM, n=5 spleen). Statistical significance was determined by Student’s *t* test (**p < 0.01; ***p < 0.001). **(B)** CK2α and β levels in CK2β^CTRL^ and CK2β^κO^ marrow and splenic purified B cells were assessed by WB. GAPDH was used as loading control (whole cell lysates; one representative of 3 independent experiments). Band quantitation was calculated using Quantity One 4.6.6 software; obtained values are reported on top of each band. **(C)** CK2 kinase activity in purified splenic B cells from CK2β^CTRL^ and CK2β^κO^ mice. Data are shown as mean ± SD (n=3 mice per genotype, 2 independent experiments). Statistical significance was determined by Student’s t test (*p < 0.05). In **(A-C)** the purity of sorted B cells was ≥94%. **(D)** p65/RELA and AKT phosphorylation in purified splenic B cells obtained by magnetic separation from CK2β^CTRL^ and CK2β^κO^ mice was assessed by WB. GAPDH was used as a loading control (whole cell lysates, one representative of 3 independent experiments). Band quantitation was calculated using Quantity One 4.6.6 software; obtained values are reported on top of each band. **(E)** Right, Scatter plots representing the percentage of CD19^+^B220^+^ cells in BM and spleen of CK2β^CTRL^ and CK2β^κO^ mice, with each symbol representing a mouse. Left, for each genotype one representative dot plot is shown, numbers in gates indicate the percentage of CD19^+^B220^+^ cells. Statistical significance was determined by Student’s *t* test (**p < 0.01). **(F)** Graphs summarizing absolute counts of B220^+^ cells in BM, spleen and PB. Data are reported as mean ± SD, statistical significance was determined by Student’s *t* test (**p < 0.01). **(G)** Bottom, Scatter plots summarizing the percentages of recirculating, immature, and Pre/Pro B cell subsets in the BM of CK2β^CTRL^ and CK2β^κO^ mice, with each symbol representing a mouse. Data are shown as mean ± SD. Statistical significance was determined by Student’s *t* test (**p < 0.01; *p < 0.05). Top, one representative contour plot per genotype shows the gating strategy. **(H)** Pro-B (CD19^+^B220^low^IgM^-^c-Kit^+^) and Pre-B (CD19^+^B220^low^IgM^-^CD25^+^) cell percentages in the BM of CK2β^CTRL^ and CK2β^κO^ mice. Data are shown as mean ± SD. Statistical significance was determined by Student’s *t* test (ns). **(I)** Bottom, Graphs representing the percentages of BM mature (B220^+^CD19^+^IgM^+/-^IgD^+^) and immature (B220^+^CD19^+^IgM^+^IgD^-^) B cells in CK2β^CTRL^ and CK2β^κO^ mice. Data are shown as mean ± SD. Statistical significance was determined by Student’s *t* test (***p < 0.001). Top, one representative dot plot is shown for each genotype. **(J)** Graphs showing percentages of T1-like (B220^+^AA4.1^+^IgM^hi^CD23^-^) and T2-like (B220^+^AA4.1^+^IgM^hi^CD23^+^) B cells in the BM of CK2β^CTRL^ and CK2β^κO^ mice. Values are reported as mean ± SD. Statistical analysis was performed with Mann-Whitney test (ns). **(K)** IgH production quantified by ELISA in the sera of CK2β^CTRL^ and CK2β^κO^ mice. Data are shown as mean ± SD (n=4). Statistical significance was determined by Student’s *t* test (**p < 0.01; *p < 0.05). BM, bone marrow.

To study the *in vivo* role of CK2β in B cell development, we generated a conditional B-cell specific KO model by crossing *Csnk2b^+/+^* or *Csnk2b^fl/fl^* to CD19-Cre transgenic mice obtaining *Csnk2b^+/+^*; CD19-Cre control (CK2β^CTRL^) and *Csnk2b^fl/fl^*; CD19-Cre KO (CK2β^KO^) mice, respectively. Analysis of B cells purified from bone marrow (BM) and spleen confirmed that CK2β^KO^ mice had markedly decreased CK2β mRNA and protein levels when compared to CK2β^CTRL^ animals (**Figures 1A, B**). Of note, we observed a stronger CK2β deletion in the spleen than in the BM, without alterations in CK2α expression (**Figure 1B**). The residue levels of CK2β in KO samples is due to the suboptimal activity of Cre recombinase in CD19-Cre mouse model; indeed, as already reported, the CD19 promoter is not 100% efficient showing 75-80% cutting capability in bone marrow-derived pre-B cells that reaches 90-95% in splenic B cells (33, 34). The decreased levels of the β subunit impair CK2α catalytic activity compromising the function of the holoenzyme, as demonstrated by *in vitro* kinase assays in splenic B lymphocytes (**Figure 1C**). To confirm the reduced catalytic capacity of CK2 we assessed the phosphorylation levels of the transcription factor p65/RelA and the kinase AKT, which are well established CK2 targets. Western blot (WB) analysis demonstrated a marked reduction in CK2-mediated phosphorylation of p65/RelA^S527^ and AKT^S129^ in CK2β^KO^ relative to CK2β^CTRL^ B cells (**Figure 1D**).

**Figure 2:**
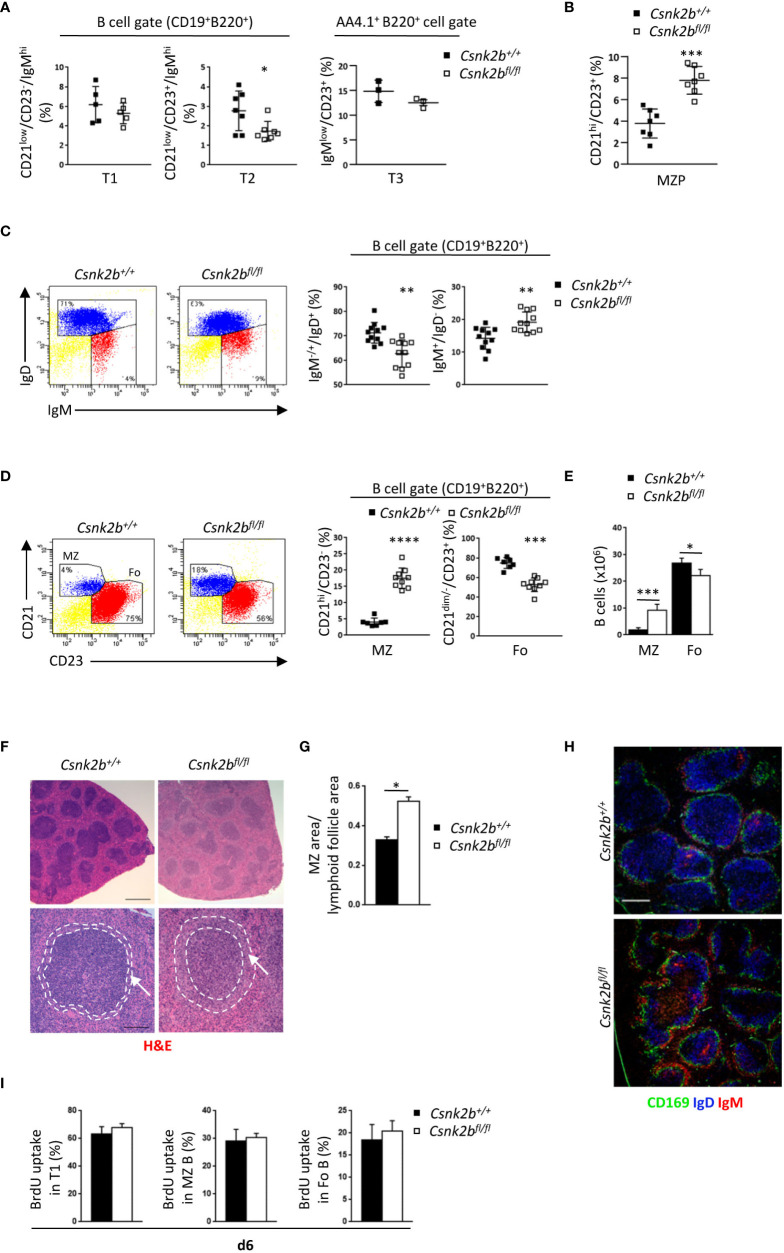
Phenotypic characterization of splenic B cell subsets in CK2β^CTRL^ and CK2β^κO^ mice. **(A)** Scatter plots summarizing the percentage of T1 (B220^+^CD19^+^CD21^low^CD23^-^IgM^hi^), T2 (B220^+^CD19^+^CD21^low^CD23^+^IgM^hi^) and T3 (B220^+^AA4.1^+^IgM^low^CD23^+^) B cells in the spleen of CK2β^CTRL^ and CK2β^κO^ mice, with each symbol representing a mouse. Statistical significance was determined by Student’s *t* test (*p < 0.05). **(B)** Scatter plots showing the percentages of MZP (CD21^hi^CD23^+^) B cells in the spleen of CK2β^CTRL^ and CK2β^κO^ mice. Data are represented as mean± SD. Statistical significance was determined by Student’s *t* test (***p < 0.001). **(C)** Right, Scatter plots representing the percentage of splenic B220^+^CD19^+^IgM^+/-^IgD^+^ and B220^+^CD19^+^IgM^+^IgD^-^ B cells of CK2β^CTRL^ and CK2β^κO^ mice, with each symbol representing a mouse. Data are shown as mean ± SD. Statistical significance was determined by Student’s *t* test (**p < 0.01). Left, one representative dot plot is shown for each genotype. **(D)** (Right) Scatter plots summarizing the percentage of MZ (B220^+^CD19^+^CD21^hi^CD23^-^) and Fo (B220^+^CD19^+^CD21^dim/-^CD23^+^) B cells in the spleen of CK2β^CTRL^ and CK2β^κO^ mice. Statistical significance was determined by Student’s *t* test (***p < 0.001; ****p < 0.0001). Left, one dot plot is presented for each genotype, numbers in gates indicate the percentages of Fo and MZ B cells. **(E)** Histogram summarizing the absolute number of Fo and MZ B cells in CK2β^CTRL^ and CK2β^κO^ mice. Data are shown as mean ± SD (n=7). Statistical significance was determined by Student’s *t* test (*p < 0.05; ***p < 0.001). **(F)** Spleen sections from CK2β^CTRL^ and CK2β^κO^ mice were stained with H&E. Bar, 500μm upper panels; 100μm lower panels. Image acquisition was performed using the Leica DMD108 Digital Microimaging Device and Software (Leica Microsystems, Germany). Data show results from one representative mouse out of 3. Arrows indicate the MZ. **(G)** Ratio between MZ and lymphoid follicle areas in the spleen of CK2β^CTRL^ and CK2β^κO^ mice was calculated using the Leica DMD108 Digital Microimaging Device and Software (2 mice per genotype; 2 independent experiments; +/+= 32 follicles; fl/fl= 54 follicles). Data are shown as mean ± SD. Statistical significance was determined by Student’s *t* test (*p < 0.05). **(H)** CD169 (green), IgD (blue) and IgM (red) expression in spleen sections from CK2β^CTRL^ and CK2β^κO^ mice was analyzed by IF. One representative mouse out of 3 per genotype is shown; 3 independent experiments. Bar, 50μm. Images were acquired with Zeiss LSM 700 confocal microscope and ZEN software. Pictures were acquired using 10x/0.3 dry and 20x/0.8 dry objectives at room temperature and merged in three-color images with ImageJ software. **(I)** Quantification of the percentage of T1, MZ and Fo B cells that incorporated BrdU after continuous administration for 6 days in CK2β^CTRL^ (n=5) and CK2β^κO^ (n=4) mice. Three independent experiments. MZ, marginal zone.

Flow cytometric analysis highlighted a significant reduction in the percentages of B cells (CD19^+^B220^+^) in BM and spleen of CK2β^KO^ mice (**Figure 1E**) that was also observed in peripheral blood (PB) and peritoneal cavity (PerC) (**Supplemental Figure 1**). However, evaluation of B220^+^ absolute cell counts evidenced no differences in BM and spleen with B lymphopenia limited to PB (**Figure 1F**). To deepen our analysis, we examined BM B-cell subsets highlighting a significant reduction in recirculating (B220^hi^IgM^+^) and an increased percentage of Pre/Pro (B220^low^IgM^-^) without changes in immature (B220^low^IgM^+^) B cells in CK2β^KO^ mice as compared to CK2β^CTRL^ animals (**Figure 1G**). Quantification of Pro-B (CD19^+^B220^low^IgM^-^Kit^+^) and Pre-B (CD19^+^B220^low^IgM^-^CD25^+^) cell subsets revealed no significant differences among genotypes (**Figure 1H**). IgM/IgD surface staining confirmed no changes in immature (B220^+^CD19^+^IgM^+^IgD^-^) B cells and a significant reduction in mature B cells (B220^+^CD19^+^IgM^+^IgD^+^) in CK2β^KO^ mice as compared to control animals (**Figure 1I**). We also investigated the features of immature Transitional (T) T1-like and T2-like B cells finding no differences between CK2β^KO^ and CK2β^CTRL^ mice (**Figure 2J**).

Remarkably, the quantification of basal serum immunoglobulin (Ig) levels revealed that all the Ig subclasses were significantly reduced in CK2β^KO^ mice as compared to CK2β^CTRL^ animals, with no substantial differences among the isotypes (**Figure 1K**). These data suggest that CK2β KO does not substantially affect BM B-cell development.

### 3.2 Decreased Fo and increased MZ B cells in CK2β^KO^ mice

The reduction in BM mature B cells and serum Ig levels in CK2β^KO^ mice, prompted us to evaluate B-cell maturation in the spleen.

We observed no variations in the percentages of transitional type 1 (T1: B220^+^CD19^+^CD21^low^CD23^-^IgM^hi^) and type 3 (T3: B220^+^ AA4.1^+^CD23^+^IgM^low^) B cells but evidenced a significant decrease in transitional type 2 (T2: B220^+^CD19^+^CD21^low^CD23^+^IgM^hi^) B cells in CK2β^KO^ as compared to CK2β^CTRL^ mice (**Figure 2A**). We then analyzed the subset of marginal zone precursors (MZP) that resulted to be expanded in the spleen of CK2β^KO^ mice (**Figure 2B**).

Moreover, CK2β^KO^ mice showed a reduction in B220^+^CD19^+^IgM^+/-^IgD^+^ cells (including T2 and Fo B cells), while the B220^+^CD19^+^IgM^+^IgD^-^ subset (including T1 and MZ B cells) was increased in comparison to CK2β^CTRL^ mice (**Figure 2C**). To gain better insights into the most mature populations, we stained splenic B cells with anti-CD23 and anti-CD21 antibodies and evidenced a significant decrease in Fo (B220^+^CD19^+^CD21^dim/-^CD23^+^) and an increase in MZ (B220^+^CD19^+^CD21^hi^CD23^-^) B cells, both in percentages and absolute numbers (**Figures 2D, E**). To deepen these results, we performed histological and immunofluorescence (IF) analyses of spleen sections. Hematoxylin and eosin (H&E) staining revealed less dense cellular lymphoid cuffs in the white pulp of CK2β^KO^ mice, which on average appeared paler than those of CK2β^CTRL^ animals (**Figure 2F**, top). MZs appeared expanded in CK2β^KO^ as compared to CK2β^CTRL^ mice (**Figure 2F**, bottom images) and we quantified a 1.6-fold increase in the *ratio* of MZ *versus* lymphoid follicle areas (**Figure 2G**). IF staining with anti-IgD, anti-CD169 (MOMA1) and anti-IgM confirmed the enlargement of the MZ, composed by the external layer of IgM^+^ B cells and the decrease in IgD^+^ Fo B cells within the follicles (**Figure 2H**). To assess if the decrease in Fo B cells could be the consequence of a reduced lifespan/proliferation of CK2β^KO^ B cells, we performed BrdU incorporation experiments *in vivo*. No differences could be detected in the percentages of BrdU^+^ cells in any splenic subpopulation (**Figure 2I**). These data suggest that in both CK2β^CTRL^ and CK2β^KO^ mice the same amount of T1 B cells migrates to the spleen, but in the latter mice these cells are more skewed towards a MZ rather than a Fo B cell fate.

### 3.3 RNA sequencing analysis highlights a critical role for CK2β in peripheral B cell activation and the germinal center reaction

To gain insights into the mechanisms underlying the Fo and MZ B cell unbalanced proportions in CK2β^KO^ animals, we performed RNA sequencing (RNAseq) on total splenic B cells purified from mice in basal condition. We identified 844 differentially expressed genes between CK2β^KO^ and CK2β^CTRL^ mice. Precisely, 445 genes were up-regulated and 399 were down-regulated in CK2β^KO^ as compared to CK2β^CTRL^ mice (**Supplemental Tables 3**, **4**).

**Figure 3:**
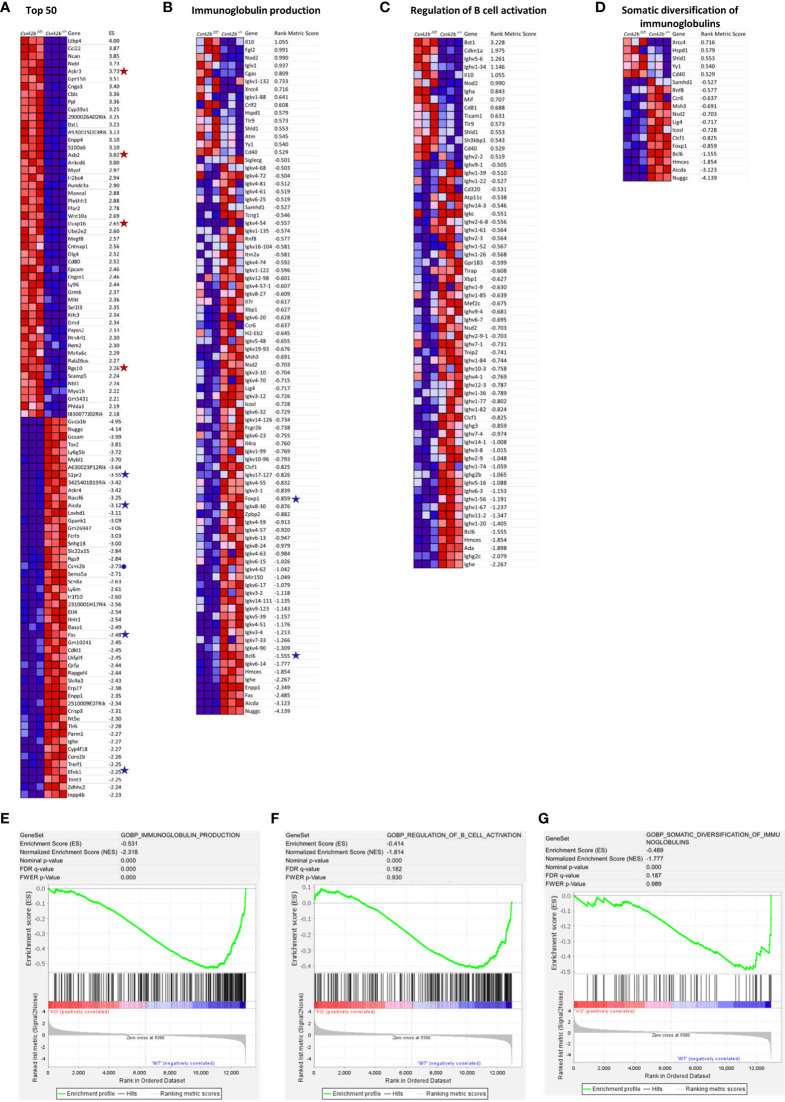
GSEA by gene set permutations of RNAseq data in CK2β^CTRL^ and CK2β^κO^ B cells in basal condition. **(A)** Heatmap of the top 50 features for each phenotype in GSE89082 as accessed by gene markers analysis. Expression values are represented as colors, where the range of colors (red, pink, light blue, dark blue) shows the range of expression values (high, moderate, low, lowest). **(B-D)** Heatmaps showing leading edge features (scores >0.5 and <-0.5) related to Gene Set GO biological process Ig production, Regulation of B-cell activation and Somatic Diversification of Igs respectively. **(E)** Enrichment score curves of significantly enriched Gene Set GO biological process Ig production. **(F)** Enrichment score curves of significantly enriched Gene Set GO biological process Regulation of B-cell activation. **(G)** Enrichment score curves of significantly enriched Gene Set GO biological process Somatic Diversification of Igs. In **(A, B)** genes of interest are flanked by a symbol: a blue dot for *Csnk2b*, red stars for over-expressed genes and blue stars for down-regulated genes in CK2β^KO^ samples. In this figure the purity of B cells isolated through sorting was >95%.

We performed gene set enrichment analysis (GSEA) on our RNAseq data. **Figure 3A** depicts the heat map of the top 50 enriched transcripts for each phenotype and their respective enrichment score in KO mice after gene set permutation analysis. This analysis confirmed qRT-PCR results (**Figure 1A**) evidencing a marked decrease in *Csnk2b* expression in KO animals (marked with a blue dot in **Figure 3A**). Interestingly, among the differentially expressed genes in CK2β^KO^ mice, *Ackr3* (also known as *Cxcr7*), a receptor for CXCL12 on MZ B cells, which promotes their retention in the MZ (35), *Asb2*, which facilitates basic helix-loop-helix transcription factors ubiquitination promoting MZ B cell formation (36), and other *bona fide* MZ B cell markers such as *Rgs10* and *Dusp16* (37, 38) (are significantly overexpressed when compared to controls (marked with a red star in **Figure 3A**). Many genes significantly downregulated in CK2β^KO^ mice when compared to control animals are relevant to GC B-cell biology (all marked with a blue star in **Figure 3A**). Among them *Aicda*, whose gene product, activation-induced cytidine deaminase (AID), initiates somatic hypermutation (SHM) and CSR (39)*, Fas*, which encodes a receptor crucial to maintain GC B-cell homeostasis (40), *Efnb1* and *S1pr2*, both important in regulating B-T cell dynamics in the GCs (41, 42). Next, we performed GO enrichment analysis and identified key biological processes significantly downregulated in CK2β^KO^ mice: “Immunoglobulin Production”, “Regulation Of B Cell Activation”, and “Somatic Diversification Of Immunoglobulins” (Heatmaps in **Figures 3B-D** and they respective GSEA enrichment plots in **Figures 3E-G**). Within these processes, multiple gene clusters of light chain immunoglobulin kappa variable region (*Igkv*) and immunoglobulin heavy chain variable region (*Ighv*) are found downmodulated in CK2β^KO^ mice, hinting towards an ineffective immune response. Of note, also *Bcl6*, a transcriptional repressor essential for the GC reaction that prevents premature activation and differentiation of GC B cells, and *Foxp1*, a transcription factor crucial for Fo and B1 B cell development and antibody (Ab) production (43) are under expressed in KO animals (both genes highlighted with a blue star in **Figure 3B**). These findings lead us to hypothesize that CK2β loss affects peripheral B cell activation by interfering with the GC reaction and by causing increased differentiation of T2 B cells into MZ B cells and decreased somatic diversification.

### 3.4 CK2β^KO^ B cells show increased activation of the NOTCH2 pathway

**Figure 4:**
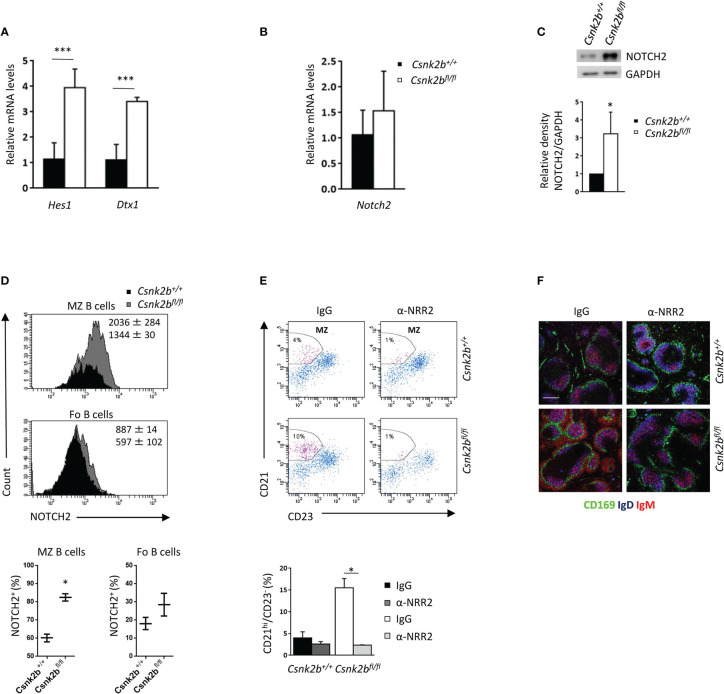
Activation of the NOTCH2 pathway determines an expansion of the MZ. **(A)** Splenic CD19^+^ B cells analyzed for the expression of *Hes1* and *Dtx1* by qRT-PCR. The expression is corrected for *Gapdh* levels and normalized to CK2β^CTRL^ B cells. Data are shown as mean ± SD (n=4, three independent experiments). Statistical significance was determined by Student’s *t* test (***p < 0.001). **(B)** Splenic B cells analyzed for the expression of *Notch2* by qRT-PCR. The expression is corrected for *Gapdh* levels and normalized to CK2β^CTRL^ B cells. Data are shown as mean ± SD (n=5, three independent experiments). **(C)** Top, NOTCH2 expression in CK2β^CTRL^ and CK2β^KO^ splenic B lymphocytes was determined by WB (whole cell lysates, one representative experiment out of three). Bottom, Mean relative density of three experiments relative to CK2β^CTRL^ B cells. Data are shown as mean ± SD. Statistical significance was determined by Student’s *t* test (*p < 0.05). In **(A-C)** the B cell fraction was purified using EasySep™ Mouse B Cell isolation kit (Stemcell) and the purity was ≥97%. **(D)** Flow cytometry analysis of NOTCH2 expression through intracytoplasmic staining. Top, NOTCH2 MFI in CK2β^CTRL^ and CK2β^KO^ mice, Bottom, graphs summarizing the percentage of NOTCH2 positive cells in the gate of MZ and Fo B cells shown as mean ± SD (two independent experiments). Statistical significance was determined by Student’s *t* test (*p < 0.05). **(E)** MZ B cells (CD21^hi^CD23^-^) from CK2β^CTRL^ and CK2β^κO^ mice were analyzed by flow cytometry after IgG or α-NRR2 administration. Top, representative dot plots indicating the MZ B cell gate. Bottom, Histograms summarizing the data of two independent experiments shown as mean ± SD. Statistical significance was determined by Student’s *t* test (*p < 0.05). **(F)** IF images of spleen sections showing CD169 (green), IgD (blue) and IgM (red) expression in IgG and α-NRR2 treated mice. Bar, 50μm. One representative mouse out of two per group is shown. Images were acquired with Zeiss LSM 700 confocal microscope and analyzed with ZEN software. Pictures were acquired using objectives 10x/0.3 dry and 20x/0.8 dry at room temperature and merged in three-color images with ImageJ software.

Since NOTCH signaling is an essential regulator of MZ B cell commitment (2), we studied the expression of its well-known downstream targets *Hes1* and *Dtx1* by qRT-PCR (**Figure 4A**). Although *Notch2* mRNA levels did not show any significant variations between CK2β^KO^ and CK2β^CTRL^ mice (**Figure 4B**), WB analysis revealed significantly higher levels of the active intracellular domain (ICD) of NOTCH2 in CK2β^KO^ as compared to CK2β^CTRL^ B cells (**Figure 4C**), suggesting post-transcriptional regulation of NOTCH2 in CK2β^KO^ B cells. By FACS analysis we observed a significant increase in NOTCH2 surface expression (measured as MFI) and NOTCH2-expressing cells in the MZ B cell compartment in CK2β^KO^ mice as compared to CK2β^CTRL^ animals (**Figure 4D**). We did not detect any increase in the mRNA levels of *Notch1* nor in the amount of the active form of NOTCH1 protein amidst the genotypes (data not shown). To demonstrate that the observed skewing towards MZ B cell differentiation in CK2β^KO^ mice is NOTCH2 dependent, we performed *in vivo* treatment with the NOTCH2 blocking antibody anti-negative regulatory region 2 (α-NRR2), which stabilizes NOTCH2 in its quiescent conformation (44). The efficacy of NOTCH2 inhibition was confirmed by qRT-PCR, which evidenced a reduction in the expression of *Hes1* and *Dtx1* in α-NRR2 treated samples (data not shown). FACS analysis proved that the blockade of the NOTCH2 pathway caused a reduction in CK2β^KO^ MZ B cells whereas treatment with non-specific IgG had no effect (**Figure 4E**). Moreover, thinning of the MZ was detected by IF analysis of CK2β^KO^ spleen sections stained for CD169, IgD and IgM after α-NRR2 or IgG treatment (**Figure 4F**). Overall, these results demonstrate that the MZ B cell compartment expansion observed in CK2β^KO^ mice is NOTCH2 dependent.

### 3.5 CK2β^KO^ mice present enlarged germinal centers upon immunization

**Figure 5:**
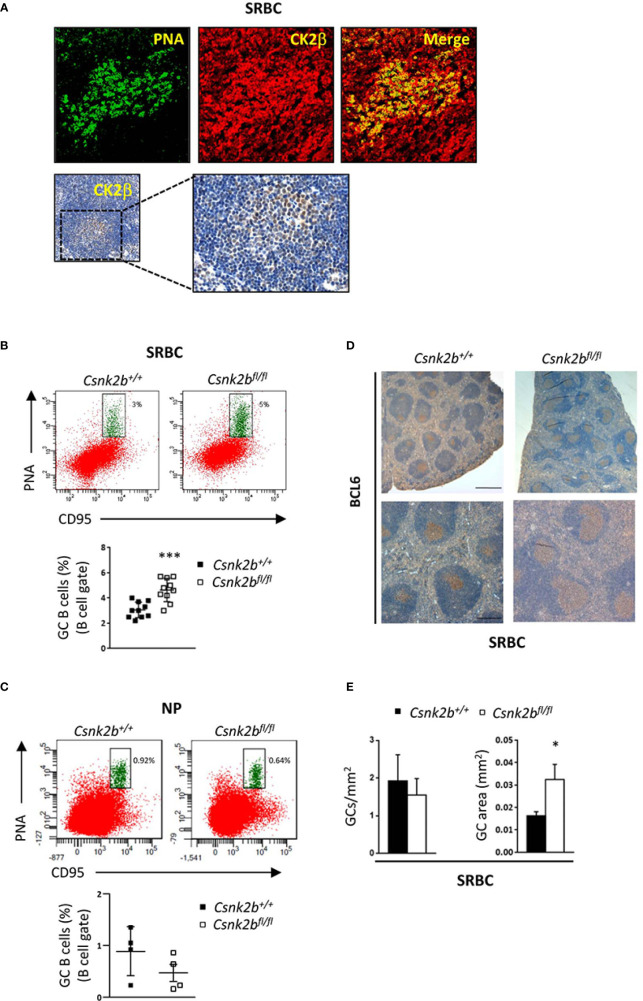
GC expansion after SRBC immunization in CK2β^κO^ mice. CK2β^CTRL^ and CK2β^κO^ mice immunized with SRBC or NP-CGG and analyzed 14 days post-immunization. **(A)** CK2β expression and localization in the GCs of CK2β^CTRL^ *s*pleens after immunization with SRBC. Top, IF images of spleen sections showing PNA (green) and CK2β (red). Images were acquired with Zeiss LSM 700 confocal microscope and analyzed with ZEN software. Pictures were acquired using objectives 20X, zoom1, and merged in two-color images with ImageJ software. Bottom, IHC of CK2β (Abcam) counterstained with hematoxylin; the magnification highlights the GC region. **(B)** GCs by Flow Cytometry in CK2β^CTRL^ and CK2β^κO^ mice after immunization with SRBC or **(C)** with NP. In **(B, C)** at the bottom, representative scatter plots of the percentage of splenic GC B cells (B220^+^CD95^hi^PNA^hi^) with each symbol representing a mouse. Data are shown as mean ± SD. Statistical significance was determined by Student’s *t* test (***p < 0.001). Top, a representative dot plot per genotype is depicted, numbers near gates indicate the percentage of B220^+^CD95^hi^PNA^hi^ cells. **(D)** GCs in spleen sections from mice of the corresponding genotypes identified through BCL6 staining and counterstained with hematoxylin. Image acquisition was performed using the Leica DMD108 Digital Microimaging Device and Software (Leica Microsystems, Germany). One representative mouse out of 3 per group is shown. Upper panels: bar, 500μm, lower panels: bar, 200μm (3 independent experiments). **(E)** Histograms summarizing the number of GCs per unit area (n=4 mice per genotype) and the area of selected GCs (n=3 mice per genotype, 7 GCs per mouse) of spleen sections stained with H&E from CK2β^CTRL^ and CK2β^κO^ mice. Data are shown as mean ± SD. Statistical significance was determined by Student’s *t* test (*p < 0.05).

Since CK2β^KO^ mice displayed a reduction in Fo B cells, we investigated the ability of CK2β-deficient B lymphocytes to efficiently mount a GC reaction and an Ab response against T-cell dependent (TD) Ags. To this aim, mice were intraperitoneally injected with sheep red blood cells (SRBCs), which are known to generate a robust immune response, or NP-CGG, which allows for the measurement of specific high-affinity Abs, and sacrificed 14 days later. IF staining of spleen sections from SRBC immunized CK2β^CTRL^ mice (top panels) and immunohistochemistry (IHC) (bottom panels) showed CK2β expression in the GCs (PNA^+^ cells), suggesting that CK2β might play a role in the GC reaction (**Figure 5A**). FACS analysis of splenic B cells from SRBC immunized mice showed higher percentages of GC B cells (CD95^hi^PNA^hi^) in CK2β^KO^ as compared to CK2β^CTRL^ mice (**Figure 5B**). No significant changes in GC B cells were instead detected between CK2β^KO^ and CK2β^CTRL^ mice upon immunization with NP-CGG likely due to the less robust response observed with this immunogen (**Figure 5C**). IHC analysis of GCs, identified through BCL6^+^ immunostaining in mice immunized with SRBCs, demonstrated that CK2β^KO^ spleens displayed larger GCs as compared to CK2β^CTRL^ samples (**Figure 5D**). In fact, while the same number of GCs per unit area was scored in both genotypes, CK2β^KO^ mice showed expanded GC areas (**Figure 5E**). These data indicate that CK2β is to some extent involved in GC progress and maturation. However, DZ and LZ B cell ratios remained unchanged in CK2β^KO^ mice when compared to controls both upon SRBC and NP-CGG immunization (data not shown).

### 3.6 CK2β deficiency impairs GC B cells class switch and plasmablasts generation

**Figure 6:**
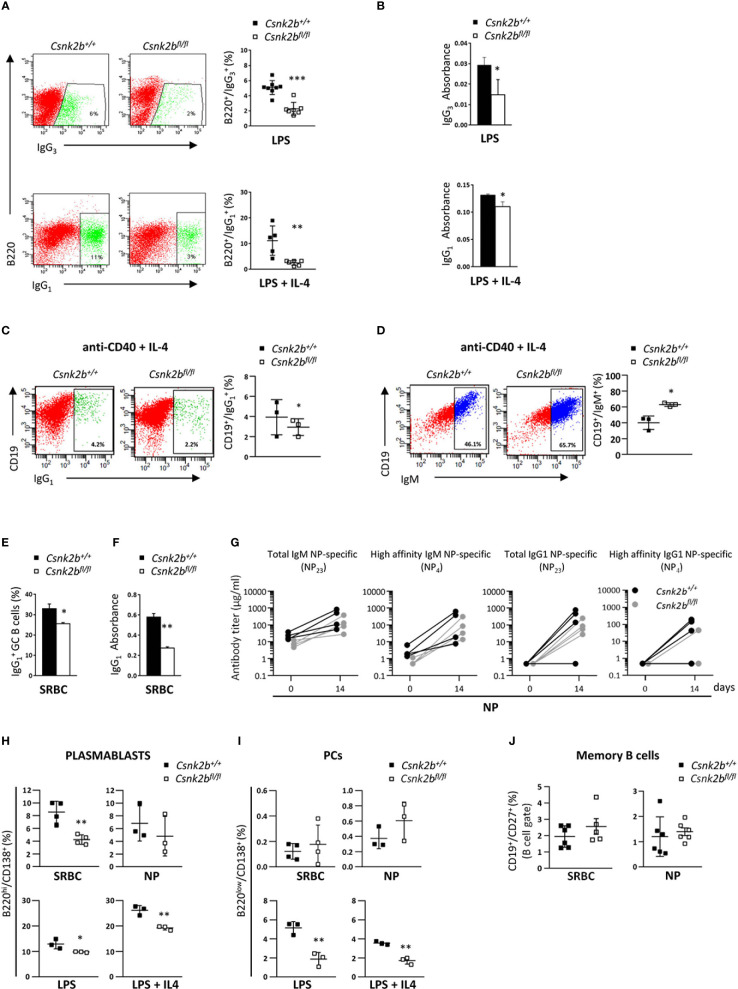
Impaired isotype-switch and Plasmablasts generation in CK2β^κO^ mice after *in vitro* stimulation and *in vivo* immunization. **(A)** CK2β^CTRL^ and CK2β^κO^ purified splenic B cells stimulated *in vitro* with LPS or LPS+IL-4 and analyzed after 72h. Right, Scatter plot of the percentage of B220^+^ ${\text{IgG}}_{3}^{+}$ or B220^+^ ${\text{IgG}}_{1}^{+}$ cells measured by Flow Cytometry, with each symbol representing a mouse. Data are shown as mean ± SD. Statistical significance was determined by Student’s *t* test (**p < 0.01; ***p < 0.001). Left, One representative dot plot per genotype is depicted. **(B)** IgG_3_ or IgG_1_ production quantified by ELISA in the cell media of CK2β^CTRL^ and CK2β^κO^ splenic B cells after exposure for 72h to LPS and LPS+IL4. Data are shown as mean ± SD (n=3 mice per genotype). Statistical significance was determined by Student’s *t* test (*, p < 0.05). **(C)** CD19^+^ ${\text{IgG}}_{1}^{+}$ and **(D)** CD19^+^IgM^+^ cells from CK2β^CTRL^ and CK2β^κO^ purified splenic B cells stimulated *in vitro* for 48h with anti-CD40+IL-4. In detail: For both **(C, D)** Right, Graphs showing the percentage of each gated population, Left, A representative dot plot per genotype. Data are shown as mean ± SD. Statistical significance was determined by Student’s *t* test (*p < 0.05). **(E)** Flow Cytometry and **(F)** ELISA in CK2β^CTRL^ and CK2β^KO^ mice after 14 days of immunization with SRBC. In detail: **(E)** Histogram summarizing the percentage of splenic IgG1^+^ GC B cells. Data are shown as mean ± SD (n=3). Statistical significance was determined by Student’s *t* test (*p < 0.05) and **(F)** IgG1 production in the serum. Data are shown as mean ± SD. Statistical significance was determined by Student’s *t* test (**p < 0.01). **(G)** Evaluation of NP-specific serum Ig titers after 14 days of NP immunization. Data were obtained through direct ELISA test, coating plates with NP23-BSA or NP4-BSA antigens to quantify total and high affinity NP-specific Abs. The concentration of total NP-specific IgM or IgG1 and high-affinity IgM and IgG1 Ab levels against NP23 and NP4 antigens are shown in Spaghetti Graphs that report Ig values before and after immunization. 4 mice per each genotype. Statistical significance was determined by Mann-Whitney U test (ns). **(H)** Graphs representing plasmablast (B220^hi^CD138^+^) percentages in the spleens of mice immunized with SRBC or NP (top) and after *in vitro* stimulation of purified splenic B cells with LSP or LPS+IL4 (bottom). Data are shown as mean ± SD. Statistical significance was determined by Student’s *t* test (*p< 0.05; **p < 0.01). **(I)** Percentages of PCs upon immunization of CK2β^CTRL^ and CK2β^KO^ mice with SRBC or NP (top) and after exposure of purified B cells to LPS or LPS+IL4 (bottom). Scatter plots indicate mean ± SD. Statistical significance was determined by Student’s *t* test (**p < 0.01). **(J)** Scatter plots showing memory B cells (CD19^+^CD27^+^) in the spleen of CK2β^CTRL^ and CK2β^κO^ mice after immunization with SRBC (left) and NP (right) for 14 days. Data are shown as mean± SD. Statistical significance is determined by Mann-Whitney test (ns). In **(A-C, H)** B cells were purified with EasySep™ Mouse B Cell Isolation Kit (Stemcell) and purity was ≥97%.

Since GSEA analysis evidenced alterations in the expression of genes involved in B cell activation and the GC reaction in CK2β^KO^ mice (**Figure 3**), and upon SRBC immunization CK2β^KO^ mice developed enlarged GCs (**Figure 5**), we investigated the impact of *Csnk2b* KO on B cell ability to perform CSR and SHM. To this aim, we stimulated CK2β^CTRL^ and CK2β^KO^ splenic B cells with lipopolysaccharide (LPS) or LPS + interleukin-4 (IL-4), which trigger IgG3 (41) and IgG_1_ CSR, respectively (42). Flow cytometric analysis showed that CK2β^KO^ B-cell cultures generated a lower fraction of IgG_3_- or IgG_1_-expressing B cells as compared to their CK2β^CTRL^ counterparts (**Figure 6A**). Quantification of IgG_3_ and IgG_1_ in the supernatant of splenic B cell cultures confirmed the reduced capacity of CK2β^KO^ B cells to secrete switched IgGs (**Figure 6B**). A similar trend was observed upon stimulation of B cells with anti-CD40 + IL-4 (**Figure 6C**). In line with this observation, in the latter condition CK2β^KO^ B cells exhibited a higher percentage of un-switched IgM^+^ B cells (**Figure 6D**). *In vitro* experiments were supported by *in vivo* data, since after SRBC immunization CK2β^KO^ mice presented a reduced percentage of splenic ${\text{IgG}}_{1}^{+}$ GC B cells and lower serum IgG_1_ levels (**Figure 6E, F**). Following immunization with NP-CGG, quantification of serum Ab titers showed a trend towards lower total and high-affinity NP-specific IgM and IgG levels in CK2β^KO^ as compared to CK2β^CTRL^ mice, yet these differences did not reach statistical significance (**Figure 6G**). The decreasing trend of high-affinity NP-specific IgG1 antibodies in serum titers of CK2β^KO^ mice prompted us to evaluate SHM in GC B cells purified by sorting (B220^hi^IgD^-^CD38^low/-^PNA^hi^CD95^+^) from spleens of NP-immunized CK2β^CTRL^ and CK2β^KO^ mice, at the peak of the immune response (14 days). Results summarized in **Table 1** report mutational analyses of the V_H_1-72 gene in B cells of three CK2β^CTRL^ and three CK2β^KO^ mice. Data are represented both as values obtained for each mouse and as the mean of mice with the same genotype. Average number of mutations within unique V gene rearrangements, and IgV_H_ mutation frequency were comparable between CK2β control and mutant groups. We also investigated the effect of CK2β deficiency on the selection within V_H_1-72 rearrangements of replacement mutations leading to the W33L amino-acid substitution, conferring a 10-fold increase in antibody binding to NP (33). With the exception of one CK2β^CTRL^ animal, which lacked the W33L amino acid substitution in any of its V_H_1-72 rearrangements, possibly due to the acquisition of complementary sets of mutations conferring similar high-affinity Ag binding properties, control animals averaged a 48% frequency of high-affinity anti-NP V gene rearrangements, in line with previous reports (45, 46). In contrast, CK2β^KO^ GC B cells showed a marked reduction in the frequency of V_H_1-72 rearrangements bearing the W33L substitution, reaching values that were roughly half of those measured in control B cells. FACS analysis also indicated a defective capacity of CK2β^KO^ mice to generate plasmablasts (PBs: B220^hi^/CD138^+^) after SRBC immunization, and after *in vitro* stimulation with LPS ± IL-4 (**Figure 6H**). No significant variations were detected upon NP immunization (**Figure 6H**). However, with the exception of B cells stimulated with LPS ± IL-4 where CK2β^KO^ developed PCs to a significantly lower extent than CK2β^CTRL^ ones, we did not observe significant differences in the percentages of PCs upon SRBC or NP immunization (**Figure 6I**). No significant differences were evidenced in the percentages of memory B cells (CD19^+^CD27^+^) in CK2β^KO^ as compared to CK2β^CTRL^ animals (**Figure 6J**). These results show that CK2β is involved in the regulation of the GC reaction and in PBs generation.

**Table 1:** Data generated from the analyses of GC B cells sorted from a total of three CTRL and three KO mice.

	Unique rearrangements/total		Average number of mutations/VH1-72 rearrangement		Mutation frequency		TGG → TTG (W33L)	
**CK2**β**^CTRL^ (CTRL)**	5/9	**23/30**	6.0	**5.41**	2.1%	**1.88%**	0.0%	**48.15%**
	9/10		6.6		2.3%		66.7%	
	9/11		3.9		1.4%		70.0%	
**CK2**β**^KO^ (KO)**	10/11	**26/34**	5.7	**5.56**	2.0%	**1.93%**	8.3%	**22.86%**
	9/10		6.7		2.3%		30.0%	
	7/13		4.7		1.6%		30.8%	

Analysis of mutations in VH1-72 V gene. Data are shown both as values per mouse and as the mean of each group of CK2β^CTRL^ and CK2β^KO^ samples. The analysis was manually performed using IgBLAST sequence analysis tool. The total amount or the mean values for each group are in bold. (Purity of sorted GC B cells ≥97%).

### 3.7 Impaired BCR signaling in CK2β^KO^ B cells

**Figure 7:**
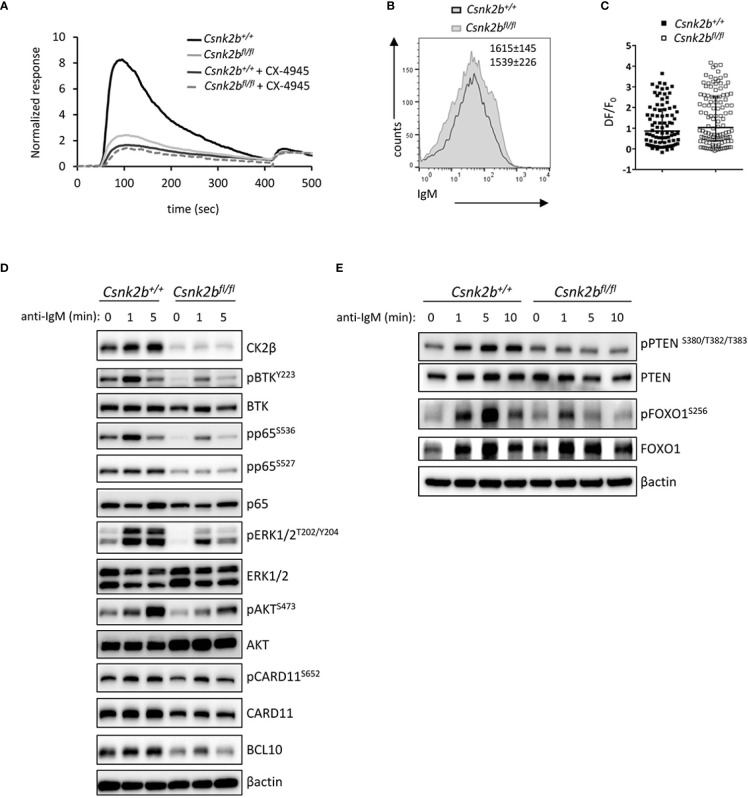
Impaired BCR signaling in CK2β^κO^ mice. **(A)** Graph representing cytosolic Ca^++^ waves in CK2β^CTRL^ and CK2β^κO^ mice ± CX-4945 pre-treatment (5μM, 3h). Cells were stimulated with α-IgM at 30 sec and ionomycin at 420 sec (n=3 CK2β^CTRL^ and 3 CK2β^κO^ mice). **(B)** Expression of IgM on the surface of B cells (CD19^+^B220^+^) from CK2β^CTRL^ and CK2β^κO^ mice was assessed by flow cytometry. Numbers on the plot show average MFI ± SD (n=6). **(C)** Scatter plot of the maximum of fluorescence variation (ΔF/F_0_) in CK2β^CTRL^ (n=86 individual cells from 2 mice) and CK2β^κO^ (n= 122 individual cells from 3 mice) B cells. Median with interquartile range is shown. Statistical significance was determined by Mann-Whitney test. **(D, E)** Study of the signaling pathways downstream of the BCR on B cells purified from the spleens of CK2β^CTRL^ and CK2β^κO^ mice treated *in vitro* for 1, 5 or 10 minutes with anti-IgM antibody. Blots are representative of four independent experiments where at least two spleens were pooled together. In **(D, E)** the B cell fraction was purified with EasySep™ Mouse B Cell Isolation Kit (Stemcell) and the purity was ≥98%.

The signaling events triggered upon BCR and NOTCH2 engagement are known to be critical for the regulation of B cell fate in the mouse (2) and are central for the initiation and maintenance of the GC reaction (47). The increase in MZ B cells, the decrease in B1 (**Supplemental Figure 2**) and Fo B cells and the up-regulation of the NOTCH2 pathway observed in CK2β^KO^ mice, suggest a likely impairment of the BCR signaling. To test this hypothesis, we stimulated purified splenic B cells with anti-IgM F(ab)_2_ Abs to trigger the BCR cascade. Firstly, as a comprehensive distal readout of BCR signaling functionality, we evaluated the intracellular Ca^2+^ flux and found a dramatic impairment in the rise of cytoplasmic Ca^2+^ concentration in CK2β^KO^ B cells. Significantly, the same impairment was observed in CK2β^CTRL^ B cells after pharmacological inhibition of CK2 kinase activity with the selective, ATP-competitive compound CX-4945 (Silmitasertib) (48, 49) (**Figure 7A**). To test if this phenomenon could be a consequence of reduced BCR surface expression in CK2β^KO^ B cells, we stained CK2β^CTRL^ and CK2β^KO^ B cells with anti-IgM Abs. However, there were no variations in the IgM surface levels between the two genotypes (**Figure 7B**). The increase in cytoplasmic Ca^2+^ levels is a consequence of its release from the endoplasmic reticulum through specific channels called inositol triphosphate (IP_3_) receptors, which bind IP_3_ produced from phosphatidylinositol 4,5-bisphosphate (PIP_2)_ after BCR stimulation (50). Many kinases phosphorylate IP_3_ receptors, inducing conformational changes that modify their capability to release Ca^2+^ in the cytoplasm (51). Since the CK2 consensus site has been identified in the IP_3_ receptor sequence (52), we argued that the reduced Ca^2+^ mobilization in CK2β^KO^ B cells could depend on impaired functionality of IP_3_ receptors. B lymphocytes were loaded with photoactivatable caged-IP_3_ and with the fluorescent Ca^2+^ indicator Fluo-4 and irradiated with an UV laser source to induce the release of IP_3_, which would bind its receptors triggering Ca^2+^ mobilization (53). We did not observe any significant difference between CK2β^CTRL^ and CK2β^KO^ B cells in response to exogenous IP_3_, suggesting that CK2β loss does not affect IP_3_ receptors conformation and functionality (**Figure 7C**).

Thus, we focused the analysis on the upstream BCR signaling cascade and proximal phosphorylation events, which lead to second messenger generation mediating Ca^2+^ mobilization (54). To this aim, B cells were incubated with anti-IgM Fab_2_ for 1, 5 and 10 minutes. The phosphorylation level of pBTK^Y223^, NF-κB pRelA/p65^S536^ and pRelA/p65^S527^, pERK1/2^T202/Y204^, pAKT^S473^ and pCARD11^S652^ were significantly reduced in CK2β^κO^ as compared to CK2β^CTRL^ B cells (**Figure 7D**). Intriguingly, we also observed reduced total CARD11 as well as BCL10 protein levels, two essential components of the CBM1 (CARD11, BCL10, MALT1) complex, which is activated by PKCβ and is essential to induce the IKK-mediated activation of NF-κB (55). An analysis of the PI3K/AKT pathway showed that the negative regulator PTEN was less phosphorylated on S^380^/T^382^/T^383^ in CK2β^κO^ B cells, a status that might correlate with an increased activity (**Figure 7E**) (56). Furthermore, as AKT-mediated phosphorylation of the transcription factor FOXO1 is a critical event in B-cell activation and function (57), we investigated FOXO1 phosphorylation levels after BCR engagement detecting markedly less pFOXO1^S256^ in CK2β^κO^ as compared to CK2β^CTRL^ B cells (**Figure 7E**).

Altogether, these results indicate a critical function for CK2β in regulating the BCR signaling cascades crucial for B-cell activation and evidence a reduced BCR strength in CK2β^κO^ mice.

### 3.8 CK2β sustains cell signaling foregoing B cell terminal differentiation

**Figure 8:**
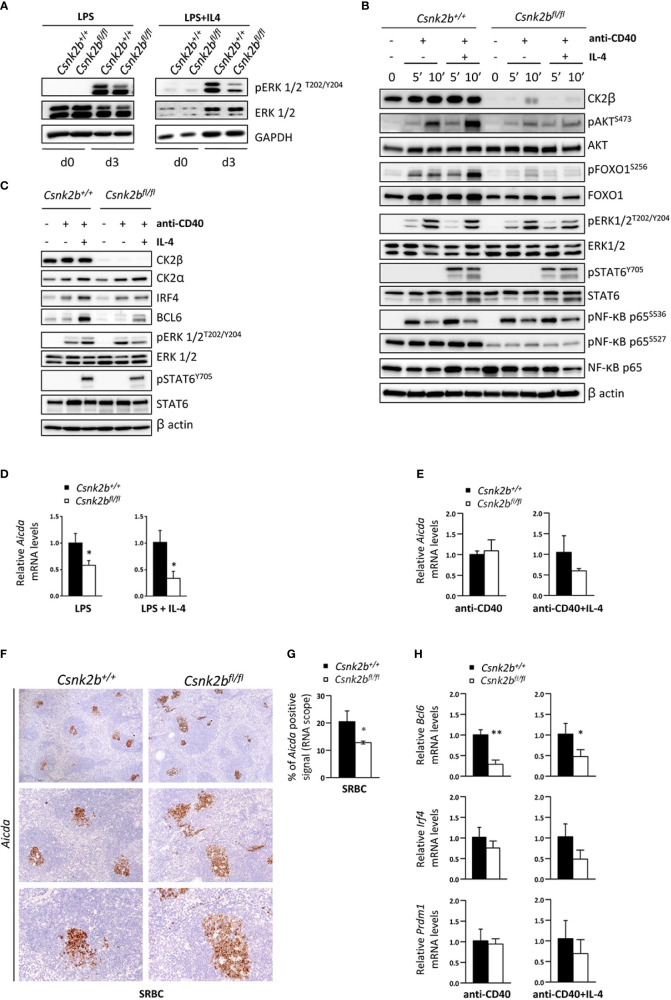
Lack of CK2β alters the activation and expression of molecules important for the GC reaction. **(A)** WB of pERK1/2 and ERK1/2 in purified splenic B cells at d=0 and d=3 of LPS and LPS+IL4 stimulation. This figure is representative of three independent experiments. **(B, C)** WB analysis of purified splenic B cells unstimulated and after anti-CD40 ± IL-4. **(B)** Stimulation for 5 and 10 minutes and **(C)** for 48 hours. Blots are representative of four independent experiments. In **(A-C)** at least two spleens were pooled together in each experiment. **(D, E)** Evaluation by qRT-PCR of *Aicda* levels in purified splenic B cells after stimulation with LPS ± IL-4 **(D)** or anti-CD40 ± IL-4 **(E)**. The expression is corrected for *Gapdh* levels and normalized to CK2β^CTRL^ B cells. Data are shown as mean ± SD (CK2β^CTRL^ n=3; CK2β^κO^ n=3). Statistical significance is determined by Student’s *t* test (*p < 0.05). **(F)** Analysis of spleen sections from CK2β^CTRL^ and CK2β^κO^ mice after immunization with SRBC (14 days) stained with RNAscope probes to detect *Aicda* levels in GCs and counterstained with hematoxylin. In order from top to bottom 4X, 10X, 20X objective magnifications. Images are representative of three controls and three CK2β^κO^ mice. **(G)** Quantitative analyses of *Aicda in situ* hybridization signals upon immunization with SRBC for 14 days. Graphs represent mean ± SD obtained by calculating the average percentage of positive signals in five non-overlapping fields for each mouse at high-power magnification using the Positive Pixel Count v9 ImageScope software, Leica Biosystems (n= 3 CK2β^CTRL^ and 3 CK2β^κO^ mice). Statistical significance is determined by Nested *t* test (*p < 0.05). **(H)** *Bcl6*, *Irf4* and *Prdm1* levels determined by qRT-PCR on purified B cells treated for 48h with anti-CD40 ± IL-4. Data were normalized over *Gapdh* and CTRL samples, and represented as mean ± SD (n=3). Statistical significance was determined by Student’s *t* test (*p < 0.05; **p < 0.01). In **(A–E, H)** the B cell fraction was purified with EasySep™ Mouse B Cell Isolation Kit (Stemcell) and purity was >97%.

To gain further insights into the role of CK2β during the GC reaction, we investigated the signaling downstream of TLR, CD40 and IL-4R, all critical pathways for B cell maturation and terminal differentiation (47). TLRs stimulated with LPS activate the MAPK-ERK1/2 pathway, which has been shown to regulate the transition from activated B cells to pre-PBs (58, 59). Immunoblot analysis after incubation of purified B cells with LPS ± IL-4 showed lower levels of pERK1/2^T202/Y204^ in CK2β^KO^ as compared to CK2β^CTRL^ B cells (**Figure 8A**). To mimic the interactions between centrocytes and follicular T helper cells in the LZ of the GCs (60), we stimulated CK2β^CTRL^ and CK2β^KO^ B cells with anti-CD40+IL-4. WB analysis was performed after early (5 and 10 minutes) as well as late (48 hours) time points. As shown in **Figure 8B**, at the early time points loss of CK2β markedly reduced the phosphorylation of pAKT^S473^ and pFOXO1^S256^ but little to no differences were detectable on pRelA^S536^ and pSTAT6^S727^. After 48h we evidenced an upregulation of CK2α in CK2β^CTRL^ cells (**Figure 8C**). We also measured the expression levels of the transcription factors BCL6 and IRF4, which are instrumental for the initiation and maintenance of-, and exit from the GC, respectively (61, 62). Immunoblot analysis indicated a reduced expression of BCL6 and IRF4 after anti-CD40+IL-4 in CK2β^KO^ as compared to CK2β^CTRL^ B cells (**Figure 8C**). As expected, BCL6 was strongly induced only by the addition of IL-4. Remarkably, in B cells from CK2β^KO^ mice, while CK2α was upregulated similarly as in CK2β^CTRL^ B cells, the rise in IRF4 and BCL6 levels after anti-CD40+IL-4 was much less pronounced (**Figure 8C**). We also checked the phosphorylation levels of ERK1/2 kinases and STAT6. To note in CK2β^KO^ B cells the level of pERK1/2^T202/Y204^ was reduced with anti-CD40+IL-4. No significant differences were seen in the levels of STAT6 phosphorylation.

We next analyzed the expression of *Aicda* (AID), the master regulator of SHM and CSR, whose transcription is upregulated upon CD40 and TLR activation (63, 64). LPS or LPS+IL-4 stimulated CK2β^KO^ B cells displayed significantly reduced levels of *Aicda* as compared to CK2β^CTRL^ B cells (**Figure 8D**). We observed a trend of reduction also upon anti-CD40+IL-4 treatment (but not with anti-CD40 alone) (**Figure 8E**). Of note, after SRBC immunization, *in situ* hybridization with specific probes showed a reduced amount of *Aicda* in CK2β^KO^ GC regions (**Figures 8F, G**). Furthermore, we analyzed the expression of the genes encoding for the transcription factors BCL6, IRF4 and BLIMP1 (*Prdm1*) upon anti-CD40 and anti-CD40+IL-4 stimulation. We observed a lower expression of *Bcl6* and a trend of decrease of *Irf4* and *Prdm1* after anti-CD40+IL-4 in CK2β^KO^ as compared to CK2β^CTRL^ B cells (**Figure 8H**). Collectively, the results indicate that CK2β positively controls SHM and CSR in GC B cells, while supporting Ag-driven selection of B cells expressing high-affinity Ab variants.

### 3.9 Chemical CK2 inhibition limits the activation of BCR pathway components in lymphoma cells

**Figure 9:**
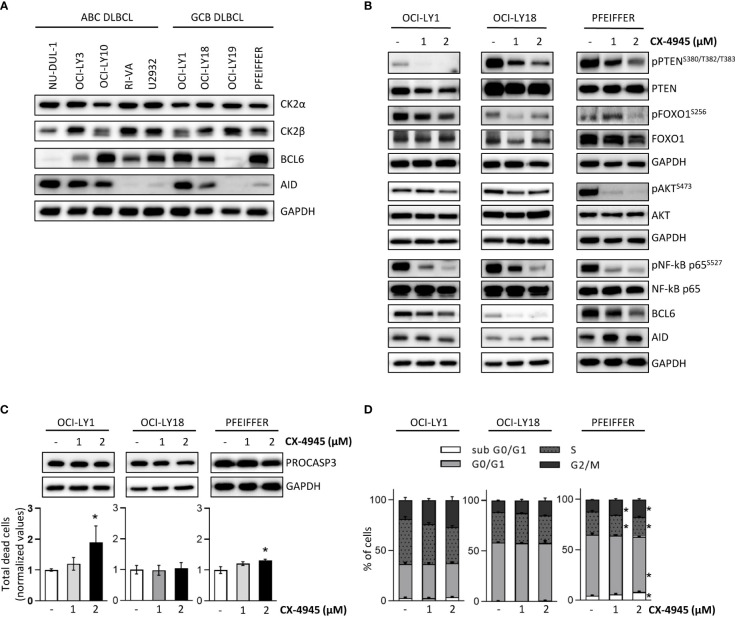
CK2 chemical inhibition reduces the activation and expression of BCR signaling molecules in DLBCL cell lines. **(A)** WB of CK2α, CK2β, BCL6 and AID baseline expression in a panel of ABC- and GCB DLBCL cell lines. **(B)** OCI-LY1, OCI-LY18 and Pfeiffer cells treated with CX-4945 (1 and 2µM) for 6 hours. **(A, B)** are representative of at least three independent experiments. GAPDH was used as a loading control. **(C)** Evaluation of OCI-LY1, OCI-LY18 and Pfeiffer cell line viability after exposure to CX-4945 (1 and 2µM) for 6 hours. Bottom, Graphs showing the percentages of dead cells after staining with AnnV/PI. Data are represented as mean ± SD of values normalized over the mean of untreated samples. Statistical significance was determined by Mann-Withney test (*p < 0.05) (OCI-Ly1 n=4; OCI-Ly18 n=5; Pfeiffer n=4). Top, representative WB of PRO-CASPASE 3 expression. GAPDH was used as a loading control. **(D)** Cell cycle analysis in OCI-LY1, OCI-LY18 and Pfeiffer cells upon treatment with CX-4945 (1 and 2µM) for 6 hours. Histograms represent the four phases of the cell cycle. Data are represented as mean ± SD. Statistical significance was determined by Mann-Withney test (*p < 0.05) (OCI-Ly1 n=4; OCI-Ly18 n=5; Pfeiffer n=4).

We and others previously claimed a potential therapeutic targeting of CK2 in malignant B cells (19, 65, 66). As we implicated CK2 in the GC reaction in healthy murine B cells, we sought to investigate the role of this kinase in malignant GC-derived Diffuse Large B Cell Lymphoma (DLBCL) cells. Firstly, we evaluated the expression levels of CK2α and CK2β, in a panel of ABC- and GCB-DLBCL cell lines. As shown in **Figure 9A**, immunoblot analysis revealed a similar expression of CK2α in all DLBCL cell lines and variable levels of CK2β. BCL6 and AID are key players in normal GC B cells maturation and BCL6 is implicated in the pathogenesis of certain subtypes of DLBCL (6, 67, 68). Immunoblot analysis indicated variable levels of BCL6 and AID expression in our panel of DLBCL cells (**Figure 9A**). To test whether CK2 inhibition impaired BCR signaling in DLBCLs, we chose three GCB-DLBCL cell lines, OCI-LY1, OCI-LY18 and Pfeiffer. These cells are BCR-dependent and present high expression of AID and BCL6. We treated these cells with CX-4945 for 6 hours. The blockade of the kinase activity caused a reduced activation of multiple BCR signaling pathway components instrumental for DLBCL survival. Similarly to what was observed in CK2β^KO^ murine B cells, we detected a prominent reduction in pPTEN^S380/T382/T383^, pFOXO1^S256^, pNF-κB^S527^ and pAKT^S473^ after exposure to the inhibitor (**Figure 9B**). Significantly, we also evidenced in all the three cell lines a notable decrease in BCL6 expression (**Figure 9B**). Since CX-4945 could affect cell viability and growth, we evaluated apoptosis through AnnexinV/PI staining (**Figure 9C**), and cell cycle through PI staining (**Figure 9D**). At the higher dose, CX-4945 caused a mild increase in apoptotic OCI-LY1 and Pfeiffer cells and a slight reduction in PRO-CASPASE 3 levels (**Figure 9C**). No differences in cell cycle progression were detected in OCI-LY1 and OCI-LY18 cells whereas Pfeiffer showed a limited increase in the subG0/G1 phase accompanied by a modest accumulation of cells in G2/M phase (**Figure 9D**). The behavior observed in Pfeiffer cells has already been reported by our group in acute myeloid leukemia-derived stem cells (69) and supports the theory of that cell cycle regulation operated by CK2 might be cell-type dependent (70). Since the observed phosphorylation and expression changes are detectable starting from the lower dose of CX-4945, we exclude that they could be due to cell toxicity triggered by CX-4945. These results, mirroring most of the alterations in signaling molecules described in CK2β^KO^ murine B cells, suggest that CK2 is essential in promoting and sustaining the activation of BCR signaling molecules also in DLBCL cells.

## 4 Discussion

In this study, we discovered a novel role for CK2β in normal B cell activation and splenic differentiation. Our results indicate that CK2β is essential for peripheral B cell development, contributes to fully proficient BCR, CD40, IL-4R and TLR signaling, and controls NOTCH2 activity.

Deletion of *Csnk2b* in CD19^+^ murine cells led to decreased CK2α kinase activity, reduced recirculating B cells and reduced serum Ab titers, the latter being likely caused to some extent by mild B lymphopenia (**Figure 1**). In line with a recent report of CK2α^KO^ in B cells (23), our CK2β^KO^ model displayed an increase in MZP and MZ B cells, as evidenced by Flow Cytometry, IF and IHC experiments (**Figure 2**). Coherently, *bona fide* MZ B cell markers *Rgs10* (71), *Ackr3* (35), *Asb2* (36) and *Dusp16* (37) are upregulated in CK2β^KO^ animals (**Figure 3**). The essential role of NOTCH2 in promoting a MZ B-cell fate is well known (2, 72) and the observation that NOTCH2 signaling inhibition rescued the MZ B cell accumulation in CK2β^KO^ (**Figure 4**) and in CK2α^KO^ animals (23) suggests that a balanced MZ B-cell expansion relies on a CK2-dependent mechanism of NOTCH2 regulation. The modulation of the splenic MZ is likely sustained by the previously described CK2-dependent phosphorylation of NOTCH2 (73) and seemingly consequent to the reduced CK2 catalytic activity.

Although CK2α^KO^ and CK2β^KO^ models show similar phenotypes, selective ablation of the catalytic rather than the regulatory subunit is not completely equivalent. Indeed, since α and β components can function in the holoenzyme but also independently from each other, differences between the two models were to be expected. Several authors have described CK2β autonomous functions, in particular as a scaffold protein essential to coordinate and/or assemble multi-protein complexes (74, 75). In detail, the β subunit has been shown to interact with and modulate the activity of a number of other serine/threonine kinases including A-Raf, c-Mos and Chk1 (76, 77). Indeed, we observed a reduction in Fo B cells in CK2β^KO^ mice that was not seen in CK2α^KO^ animals. Since only the loss of β results in decreased levels of Fo B cells, we hypothesize that this regulation might derive from the above-mentioned α-independent functions of CK2β in regulating additional kinases presumably involved in Fo B cell maintenance. In addition, considering that the absolute counts of splenic B cells are not altered in KO mice when compared to control animals, the reduced amount of Fo B cells is to be attributed to the observed differences in BCR signaling strength and NOTCH2 activation (**Figures 4**, **7**).

It has been reported that in basal conditions, commensal microbial population could play a role in the basal stimulation of the immune system and that mice in a pathogen free environment display a fixed number of preexisting niches that are formed by follicular dendritic cells and contain a small amount of GC B cells (78). Indeed, GSEA and GO biological processes enrichment analysis on RNAseq data from purified spleen B cells highlighted in CK2β^κO^ B cells a down-regulation of genes involved in GC biology, regulation of B cell activation and Ig production already at baseline (**Figure 3**).

Indeed, when using a strong immunizing agent, SRBC, CK2β^KO^ GCs were significantly expanded when compared to controls (**Figure 5**). This aberrant accumulation of GC B cells was previously described upon Fas deletion (40) and is consistent with the decreased *Fas* expression observed in CK2β^KO^ mice (**Figure 3A**). Moreover, results from *in vitro* stimulation and *in vivo* immunization experiments indicated that B cells lacking CK2β are less efficient in CSR and in completing NP-specific high affinity maturation through SHM as well as impaired in the capability to generate short-lived PBs (**Figure 6**). However, only *in vitro* data highlighted differences in the percentages of PCs between KO and control mice. This discrepancy between *in vivo* and *in vitro* results is likely to be partly attributed to the existence of a follicular immune microenvironment comprising T regulatory, T helper and dendritic cells able to trigger PCs generation, which is absent in *in vitro* settings (79). The evidenced differences between PBs and PCs in CK2β^KO^ mice could be the consequence of potentially different origins of the two populations. While PCs mainly develop from B cells passing through the GC, PBs can also have an extra-follicular origin (80).

In line with GSEA and GO enrichment analysis, CK2β^KO^ B cells displayed lower B cell activation due to impaired signal transduction downstream of multiple surface receptors (**Figures 7**, **8**). Ca^2+^ mobilization after anti-IgM stimulation was severely defective in KO cells. The altered Ca^2+^ mobilization upon BCR activation is attributable to upstream signaling defects, as suggested by the flawed phosphorylation of AKT, ERK and p65/RelA in CK2β^κO^ B cells. The reduction in BCR signaling strength, being permissive for NOTCH2 activation, contributes to the skewed differentiation towards a MZ B cell phenotype in CK2β^KO^ mice. In further support of this notion, we found a reduction in B1 B cells, a population developing from fetal liver progenitors (81) that relies on a strong BCR signaling for its development (3, 82). In CK2β^KO^ B cells, acute stimulation with CD40 + IL-4 was associated with an impaired AKT and, to a lower extent, ERK1/2 activation (**Figure 8**). Upon *in vitro* stimulation, we observed a compromised PI3K/AKT signaling in CK2β^KO^ B cells with the expected activation of its downstream target FOXO1. Prolonged TLR, CD40 and IL-4R stimulation evidenced a markedly reduced upregulation of critical controllers of the GC reaction and PC development, such as the transcription factors BCL6 and IRF4 in CK2β^KO^ B cells (**Figures 8C**). Another critical finding, in line with RNAseq data, was the lower *Aicda* level detected in CK2β^KO^ B cells after LPS stimulation and SRBC immunization (**Figures 8D–G**). Given the central role of AID in CSR, SHM and affinity maturation of Ag-engaged B cells (83), these findings could in part explain why CK2β^KO^ mice are not able to efficiently produce class-switched Ig and complete Ab affinity maturation, as demonstrated by the reduced frequency of W33L point mutation in VH186.2 genes upon NP immunization (**Table 1**).

The signaling changes seen in normal murine B cells after CK2β loss prompted us to analyze the consequences of CK2 inactivation in malignant GC-derived B cells. In DLBCL cells, CK2α inhibition caused a reduction in the phosphorylation of AKT, FOXO, and p65/RelA, combined with decreased BCL6 expression (**Figure 9B**), similarly to what was observed in normal murine B cells (**Figures 8B**). Together with previously described findings of CK2 upregulation in various cancers, this data supports the hypothesis that, although CK2 by itself is not an oncogene, it might have a role in sustaining the process of malignant transformation, proliferation and survival as previously argued in this and other hematologic malignancies (16, 19, 21, 66, 84–87).

In conclusion, our work has shown that CK2β orchestrates mature B cell fate and is involved in the regulation of the GC reaction by acting at multiple levels in the B cell signaling network. Although the signaling defects observed in KO animals are likely dependent on the impaired activity of the CK2 holoenzyme, we hypothesize that an α-independent role of CK2β is at the basis of the observed reduction in Fo B cells. Further studies are needed to elucidate CK2α-dependent and independent effects in the observed phenotype. Additionally, we also demonstrate that CK2-dependent signaling is exploited by malignant B cells for proliferation and survival.

## Data availability statement

The datasets presented in this study can be found in online repositories. The names of the repository/repositories and accession number(s) can be found below: Gene Expression Omnibus (http://www.ncbi.nlm.nih.gov/geo) under accession number GSE89082.

## Ethics statement

The animal study was reviewed and approved by Italian Ministry of Health (Prot. number 126/2015 and Prot. 205/2022).

## Author contributions

FP devised the study. EM, LQT, SCN, AA, FZ, SM, AC, SZ and PM performed the experiments. EM, LQT, FZ, AC, SM, SZ, and PM developed methodology. AV helped designing the calcium study. OFC and BB provided the mice. MP and AD performed immunohistochemistry. NV and GV performed the RNAseq. SC and FM designed and contributed to execute experiments to analyze germinal center B cell responses. VC, AG, and CT performed RNA scope. EM, LQT, SCN, FZ, AC, FP, MP, SZ, NV, and PM analyzed data and made the figures. CWS provided α-NRR2 reagent. LQT, EM, SCN, SC, FM and FP wrote the manuscript. LT, GS, SM, SC and FP provided funding. All authors contributed to the article and approved the submitted version.
