# Supplementary material for: CK2β-regulated signaling controls B cell differentiation and function
Source: Front Immunol. 2023 Jan 11;13:959138. doi: 10.3389/fimmu.2022.959138 (PMC9874936; doi:10.3389/fimmu.2022.959138)
Supplement: Supplementary file 1 [file DataSheet_1.pdf]

Supplementary Material

Supplementary Methods

Direct ELISA test against NP-23 and NP-4 antigens. NP_{23}-CGG and NP_4-CGG antigens supplied as powder 10mg were resuspend in 1 ml of PBS (10 mg/ml). NP_{23}-BSA Antigen, NP_4-BSA Antigen or BSA as control were then diluted at the working concentration of 2 mg/ml in Carbonate-Bicarbonate 0.5mM Coating Buffer 0.2 mm filtered. For NP4 Standard curve coating was performed with Capture Antibody (CA) (LO-MG-13 ABD-Serotec MCA1289, rat anti-mouse IgG1, IgG1) diluted to the working concentration of 1mg/ml in Carbonate-Bicarbonate 0.5mM Coating Buffer 0.2 mm filtered. A NUNC Maxisorp white 96 well microplate with 50 ml per well was immediately coated with the dilute CA. The plate was sealed and incubated overnight at 4°C or 3 hours at 37°C. Three washes were performed with washing buffer (PBS-Tween20 0,05%) followed by blocking the plate by adding 100 ml per well of Blocking Buffer (3% BSA in PBS) Plate was incubated at 37°C for one hour followed by two washes. 50 µl of sample or NP specific antibody N1G9 or Mouse Reference Serum were added in standard wells in Reagent Diluent (RD) (1% BSA in PBS). Plate was sealed and incubated 1 hour at 37°C or overnight at 4°C and then washed four times with washing buffer. To each well 50 µl of the Detection Antibody IgG1 (BD Pharmingen 550331, 500 mg/ml of biotinylated anti-mouse IgG1) was added in Reagent Diluent to the working concentration of 125 ng/ml; plate was covered and incubated 1 hour at room temperature followed by five washes. 50 µl of the Streptavidin-Eu^{3+} (Perkin Elmer) diluted in reagent diluent was added to each well. The plate was covered and incubated for 30 minutes at room temperature in the dark followed by six washes. Finally, 50 µl of room temperature DELFIA Enhancement Solution (4001-0010 Perkin Elmer) were added to each well and incubation was performed in the dark at room temperature for 15 minutes, shaking slowly. Fluorescence was detected with time-resolved fluorometer Victor3.

Supplementary Figures

Figure S1

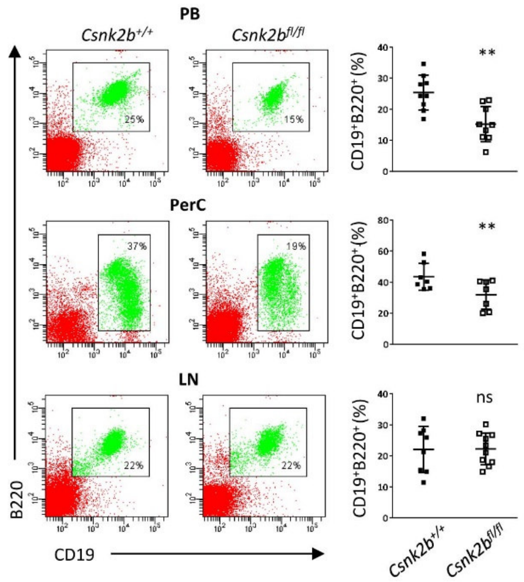

Supplementary Figure 1. Flow cytometry analysis of B cells in PB, PerC and LN. PB, PerC and LN cell suspensions were washed with PBS 1X and $0.5x10^5$ cells were stained with CD19-PerCP-Cy5.5 (1D3) and B220-APC-Cy7 (RA3-6B2) Abs, BD. Left: one representative dot plot per genotype is shown. Right: scatter plot of the percentage of $CD19^+B220^+$ cells. Data are shown as mean ± SD (*, $p < 0.05$; **, $p < 0.01$).

Figure S2

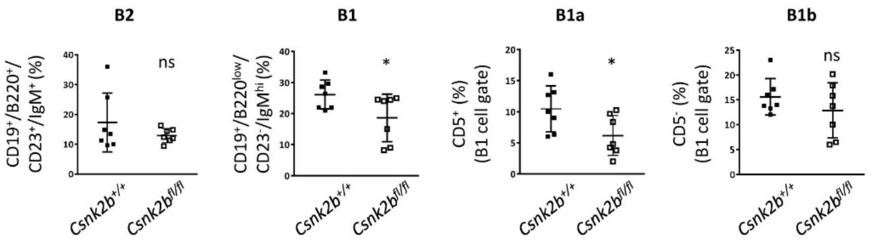

Supplementary Figure 2. Flow cytometry analysis of B1 and B2 B cells in PerC. PerC cells were collected washing the PerC with 3 ml of PBS 1X and stained with CD19-PerCP-Cy5.5 (1D3), CD23-PE (B3B4), IgM-PE Cy7 (R6-60.2), CD5-FITC (53-7.3) and B220-APC-Cy7 (RA3-6B2) Abs, all BD. Data are shown as mean ± SD (*, $p < 0.05$). B1 cell gate= CD19^{+}B220^{low}CD23-IgM^{hi}.

Supplementary Tables

Supplementary Table 1. Primers used for qRT-PCR analyses on murine B cells.

Gene	Forward	Reverse
Csnk2b	5'-AGAGCTGGAAGACAACCCCAA-3'	5'-CCAACATTTGTGCGATGCC-3'
Gapdh	5'-CACCATCTTCCAGGAGCGAG-3'	5'-CCTTCTCCATGGTGGTGAAGAC-3'
Notch2	5'-ATGTGGACGAGTGTCTGTTGC-3'	5'-GGAAGCATAGGCACAGTCATC-3'
Hes1	5'-CCAGCCAGTGTCAACACGA-3'	5'-AATGCCGGGAGCTATCTTTCT-3'
Dtx1	5'-CATCAGTTCCGGCAAGAC-3'	5'-GATGGTGATGCAGATGTCC-3'
Actin	5'-CTCTCCCTCACGCCATCCTG-3'	5'-TCACGCACGATTTCCCTCTCA-3'
Aicda	5'-AGGGAGTCAAGAAAGTCACG-3'	5'-CAGGAGGTGGCACTATCTCT-3'
Bcl6	5'-GACTGTCCACACGGGTGAGA-3'	5'-GGGCCACCTGAACAAACCT-3'
Irf4	5'-TCACGAGGATGTCCCGGTAA-3'	5'-GAAGCCTTGGCGCTCTCA-3'
Prdm1	5'-CCCTCTGAAGAAACAGAATG-3'	5'-GCTTGTGCTGCTAAATCTCT-3'

Supplementary Table 2. Primers for V_H186.2 semi-nested PCR amplification.

V186.2 outer primer Forward	5'-TCTTTACAGTTACTGAGCACACAGGAC-3'
JH2 Reverse	5'-GGGTCTAGAGGTGTCCCTAGTCCTTCATGACC-3'
V186 inner primer Forward	5'-CAGTAGCAGGCTTGAGGTCTGGAC-3'
